# IL-27 triggers IL-10 production in Th17 cells via a c-Maf/ROR*γ*t/Blimp-1 signal to promote the progression of endometriosis

**DOI:** 10.1038/cddis.2017.95

**Published:** 2017-03-16

**Authors:** Kai-Kai Chang, Li-Bing Liu, Li-Ping Jin, Bing Zhang, Jie Mei, Hui Li, Chun-Yan Wei, Wen-Jie Zhou, Xiao-Yong Zhu, Jun Shao, Da-Jin Li, Ming-Qing Li

**Affiliations:** 1 Laboratory for Reproductive Immunology, Hospital of Obstetrics and Gynecology, Fudan University, Shanghai 200011, People's Republic of China; 2 Key Laboratory of Reproduction Regulation of NPFPC, SIPPR, IRD, Fudan University, Shanghai 200032, People's Republic of China; 3 Shanghai Key Laboratory of Female Reproductive Endocrine Related Diseases, Shanghai 200011, People's Republic of China; 4 Clinical and Translational Research Center, Shanghai First Maternity and Infant Hospital, Tongji University School of Medicine, Shanghai 200040, People's Republic of China

## Abstract

Endometriosis is an estrogen-dependent inflammatory disease. The anti-inflammatory cytokine IL-10 is also increased in endometriosis. IL-10 production by Th17 cells is critical for limiting autoimmunity and inflammatory responses. However, the mechanism of inducing IL-10-producing Th17 cells is still largely unknown. The present study investigated the differentiation mechanism and role of IL-10-producing Th17 cells in endometriosis. Here, we report that IL-10^+^Th17 cells are significantly increased in the peritoneal fluid of women with endometriosis, along with an elevation of IL-27, IL-6 and TGF-*β*. Compared with peripheral CD4^+^ T cells, endometrial CD4^+^ T cells highly expressed IL-27 receptors, especially the ectopic endometrium. Under external (2,3,7,8-tetrachlorodibenzo-p-dioxin, TCDD) and local (estrogen, IL-6 and TGF-*β*) environmental regulation, IL-27 from macrophages and endometrial stromal cells (ESCs) induces IL-10 production in Th17 cells *in vitro* and *in vivo*. This process may be mediated through the interaction between c-musculoaponeurotic fibrosarconna (c-Maf) and retinoic acid-related orphan receptor gamma t (ROR*γ*t), and associated with the upregulation of downstream B lymphocyte-induced maturation protein-1 (Blimp-1). IL-10^+^Th17 cells, in turn, stimulate the proliferation and implantation of ectopic lesions and accelerate the progression of endometriosis. These results suggest that IL-27 is a pivotal regulator in endometriotic immune tolerance by triggering Th17 cells to produce IL-10 and promoting the rapid growth and implantation of ectopic lesions. This finding provides a scientific basis for potential therapeutic strategies aimed at preventing the development of endometriosis, especially for patients with high levels of IL-10^+^Th17 cells.

As the most common gynecological disease in women, endometriosis (EMS) affects approximately 10% of women during their reproductive years. Its defining feature is the presence of endometrium-like tissue in sites outside the uterine cavity, primarily on the pelvic organs. This disease is mainly associated with pain, including dysmenorrheal, deep dyspareunia and infertility. Despite decades of investigation, little is known about the pathogenesis of EMS. The most widely accepted etiology is Sampson's theory of retrograde menstruation. That is, shed endometrial tissue is refluxed through the fallopian tubes and proliferates and implants within the pelvis.^1^ However, the theory cannot fully explain that while the majority of women have retrograde menstruation, only about 1 in 10 women develops EMS.

A distorted immune response against endometrial cells not only cannot effectively remove endometrial debris in the pelvic cavity from menstrual blood flow, but also can facilitate its implantation, neoangiogenesis and proliferation. However, the underlying mechanism is largely understood. In addition to pro-inflammatory cytokines, such as TNF-*α*, IL-1*β* and IL-17,^2, 3, 4^ it has yet to be reported that anti-inflammatory cytokine IL-10 is increased in the peritoneal fluid (PF) and/or peripheral blood of women with EMS, especially in advanced stages.^3, 5, 6, 7, 8^ Our previous work identified IL-10 produced by regulatory T (Treg) cells as a key mediator in regulating the growth and implantation of ectopic endometrial tissue.^7^

Of note, IL-10 regulation in the adaptive T-cell response is more complex, with multiple Th2-independent pathways. IL-27 promotes Stat1-dependent IL-10 production in both Th1- and Th2-polarizing conditions.^9, 10^ TGF-*β* is synergistic with both IL-6 and IL-27 for c-Maf expression and consequent IL-10 production in mouse Th17 cells, which further restrains the pathologic effects of Th17 cells.^11, 12^ Despite these findings in mouse Th17 cells, however, it is unclear whether human IL-10-producing Th17 cells exist in the PF from EMS patients and share features with these cells in mouse.

IL-27 is produced by activated antigen-presenting cells (APC).^13^ It promotes T helper (Th) 1 and type 1 regulatory T (Tr1) cells, but inhibits Th2, Th17 and Treg cell differentiation and function. Under certain conditions, opposite effects on certain T-cell subsets have been observed. IL-27 displays pro- or anti-inflammatory activity in different autoimmune diseases.^14, 15, 16^ However, the precise condition that controls the dual functional attributes of IL-27 has not been fully defined. In this study, we aimed to characterize the role of IL-27 in the endometriotic milieu regarding IL-10-producing Th17 cell differentiation and EMS progression.

## Results

### IL-10^+^Th17 cells in the endometriotic milieu were gradually elevated with the progression of EMS

**Figure 1:**
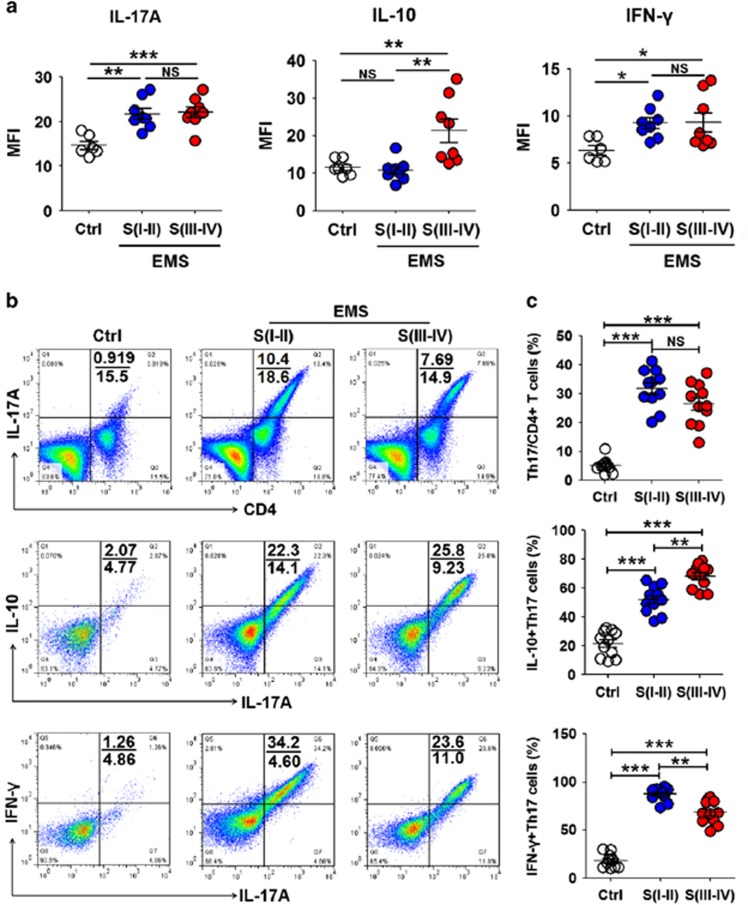
IL-10^+^Th17 cells in the endometriotic milieu were gradually elevated with the progression of EMS. (**a**) IL-17A, IL-10 and IFN-*γ* levels in peritoneal fluid (PF) from women with or without endometriosis by CBA assay (one-way ANOVA). (**b** and **c**) The percentage of Th17 cells, IL-10^+^Th17 cells and IFN-*γ*^+^Th17 cells in PF from women with or without endometriosis by flow cytometry (one-way ANOVA). Ctrl, PF from women without endometriosis (*n*=6); EMS S(I–II), PF from women with endometriosis in early stages (stage I and II, *n*=8); EMS S(III–IV), PF from women with endometriosis in advanced stages (stage III and IV, *n*=8). The data are expressed as the mean±S.E.M. *P<0.05, **P<0.01 and ***P<0.001; NS, no statistical difference

We first examined the cytokine profile of the PF in patients with EMS. The pro-inflammatory cytokines such as IFN-*γ*, TNF-*α*, IL-1*β* and IL-17A, were increased in patients with stage I–II disease, but there was no more elevation with the progression of EMS (Figure 1a and Supplementary Figure 1). Significant increases of anti-inflammatory cytokines IL-10 and IL-4 were limited to patients with stage III–IV disease (Figure 1a and Supplementary Figure 1). As we observed the elevation of key cytokines IL-6 and TGF-*β* for Th17 cell differentiation (Supplementary Figure 1) in the PF with EMS, we next sought to investigate Th17 cell levels and found that the percentage of Th17 cells in CD4^+^ T cells from the PF in patients with stage I–II disease was increased to 31.8% (Figures 1b, c, Supplementary Figures 2A and B). However, IL-10^+^Th17 cells reached a peak, and IFN-*γ*^+^Th17 cells were slightly decreased in patients with Stage III–IV disease (Figures 1b and c). These results indicated that elevated IL-10 in patients with advanced EMS is derived from Th17 cells. With the progression of EMS, some Th17 cells may undergo global genetic reprogramming to drive their conversion from a pro-inflammatory phenotype to an anti-inflammatory phenotype.

### Internal and external environments lead to an accumulation of IL-27 in the endometriotic milieu

**Figure 2:**
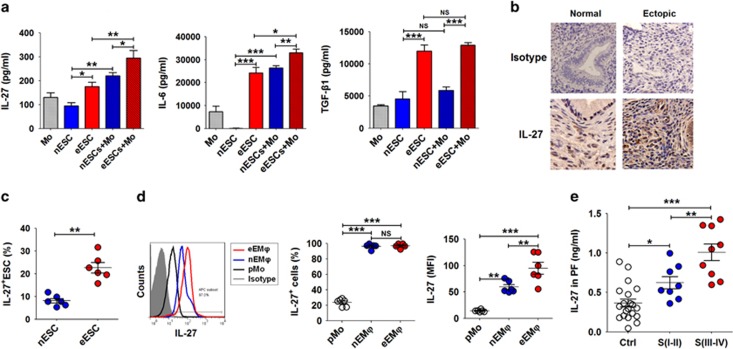
ESCs and macrophages from endometriotic lesions highly expressed IL-27. (**a**) We co-cultured ESCs (normal ESCs or ectopic ESCs) with monocytes (*n*=5) from peripheral blood for 48 h. In addition, ESCs alone and monocytes alone were cultured as controls. Then, the secretion levels of IL-27, IL-6 and TGF-*β*1 were analyzed by ELISA. nESCs, ESCs from normal endometrium; eESCs, ectopic lesion with endometriosis; nESCs+Mo, co-culture of nESCs and monocytes; eESCs+Mo, co-culture of eESCs and monocytes (one-way ANOVA). (**b**) IL-27 expression in normal endometrium (*n*=6) and ectopic endometrium from women with endometriosis (*n*=8) by immunohistochemistry. Normal, normal endometrium; ectopic lesion from women with endometriosis. Original magnification: × 200. (**c**) The percentage of IL-27^+^ESCs (normal ESCs or ectopic ESCs, *n*=6) by flow cytometry (Student's *t*-test). (**d**) The percentage and median fluorescence intensity (MFI) of IL-27^+^monocytes of peripheral blood (pMo, *n*=6) and IL-27^+^macrophages of normal endometrium (nEM*ϕ*, *n*=6) and ectopic lesions (eEM*ϕ*, *n*=6) by flow cytometry (one-way ANOVA). (**e**) The IL-27 level in PF from women with (*n*=17, I–II: 8; III–IV: 9) and without (*n*=24) endometriosis by ELISA (one-way ANOVA). The data are expressed as the mean±S.E.M. *P<0.05, **P<0.01 and ***P<0.001

Macrophages were the highest population (approximately 60%) in PF leukocytes from EMS patients (Supplementary Figures 2A and B). To identify the key regulatory factors driving Th17 cells towards regulatory states (IL-10^+^Th17 phenotype) in the endometriotic milieu, a co-culture model with primary ESC (Supplementary Figure 3) and peripheral blood monocytes was constructed to imitate the ectopic immune microenvironment of EMS. Compared with normal ESC, the secretion level of monocyte chemotactic protein-1 (MCP-1), CCL5 (also known as RANTES) and granulocyte–macrophage colony-stimulating factor (GM-CSF) by ectopic ESCs was markedly upregulated (Supplementary Figure 4). Co-culture with monocytes led to higher productions of MCP-1, RANTES and GM-CSF, which may be involved in an infiltration accumulation of monocytes from the peripheral tissue to the ectopic lesion and monocyte-to-macrophage differentiation and maturation. In addition, ectopic ESCs secreted higher levels of IL-27, IL-6 and TGF-*β*, especially in the co-culture system (Figure 2a). In addition to ectopic ESCs (Figures 2b and c), nearly 100% of macrophages in both the normal and ectopic endometrium expressed IL-27 (Figure 2d). However, there were 1.60-fold and 6.56-fold increases of IL-27 fluorescence intensity by macrophages in ectopic endometrium compared with that in normal endometrium and peripheral blood (Figure 2d), respectively. The results were similar in mouse (Supplementary Figure 5). Interestingly, similarly with IL-10^+^Th17 cells, IL-27 in the PF was also gradually accumulated with EMS progression (Figure 2e).

**Figure 3:**
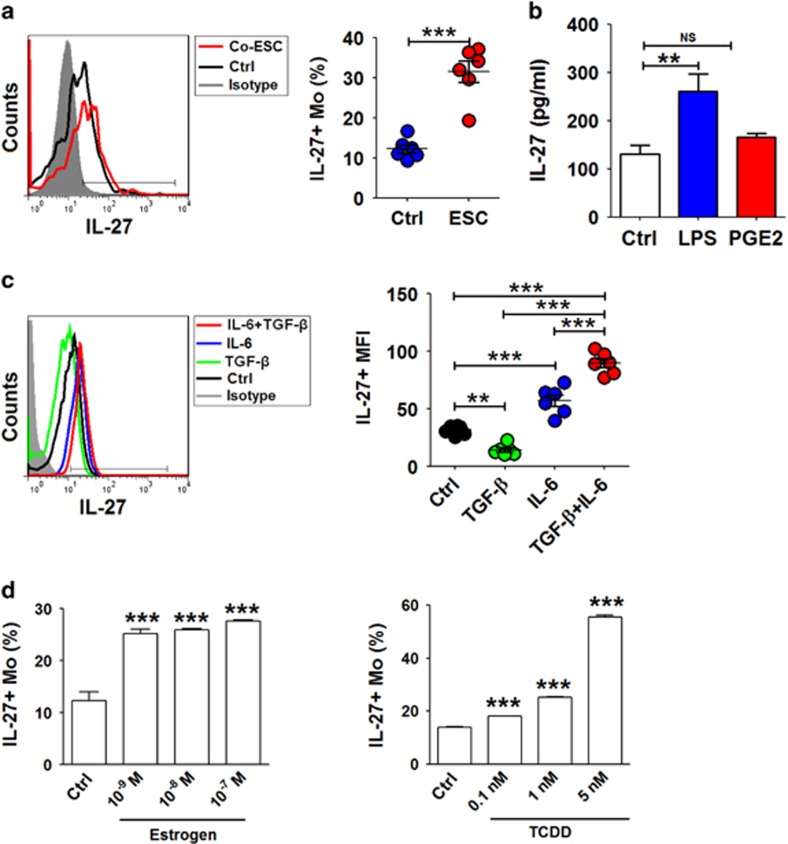
Internal and external environments lead to an accumulation of IL-27 in macrophages from endometriotic lesions. (**a**) The percentage of IL-27^+^monocytes from peripheral blood (*n*=6) co-cultured with or without ESCs for 48 h by flow cytometry. Ctrl, monocytes alone; ESCs, monocytes co-cultured with normal ESCs (Student's *t*-test). (**b**) The monocytes (*n*=6) were stimulated with LPS (10 ng/ml) or PGE2 (10^−6^ M) for 48h, and then IL-27^+^ monocytes was analyzed by flow cytometry (one-way ANOVA). (**c**) Recombinant human TGF-*β* (rhTGF-*β*, 5 ng/ml) and or IL-6 (rhIL-6, 50 ng/ml) was added to an ESC-monocyte co-culture system (*n*=6) for 48 h, and then IL-27^+^ monocytes were analyzed by flow cytometry (one-way ANOVA). (**d**) After treatment with 17*β*-estrogen (10^−9^–10^−7^ M) or TCDD (0.1–5 nM), the percentage of IL-27^+^ monocytes was analyzed by flow cytometry (one-way ANOVA). The data are expressed as the mean±S.E.M. **P<0.01 and ***P<0.001 *versus* control group

Endometrial debris in the pelvic cavity from menstrual blood flow is in direct contact with macrophages and other leukocytes in the PF recruited from the peripheral tissue, and the interaction between these cell subsets has an important role in maintaining the growth and implantation of ectopic endometrium.^17, 18, 19, 20^ The co-culture with ESCs led to a threefold upregulation of IL-27^+^ monocytes (Figure 3a). In addition, LPS stimulation significantly increased IL-27 levels in monocytes (Figure 3b). TGF-*β* alone resulted in the decrease of IL-27 while it synergistically upregulated IL-27 in monocytes with IL-6 (Figure 3c). This combined effect of multiple factors in the peritoneal cavity or ectopic lesion led to an increased level of IL-27 by macrophages in endometriosis.

Here, we observed that estrogen (10^−9^ M) increased IL-27 secretion (Figure 3d). The incidence and severity of EMS are also associated with exposure to TCDD.^21^ Treatment with TCDD induced an approximate fivefold upregulation of IL-27 in monocytes (Figure 3d). These data suggest that aberrantly high levels of IL-27 may be a synthetic result of multiple factors, including the cross-talk between ESCs and macrophages, high local estrogen stimulation and TCDD exposure.

### IL-27 induces IL-10 production of Th17 cells in the endometriotic milieu

**Figure 4:**
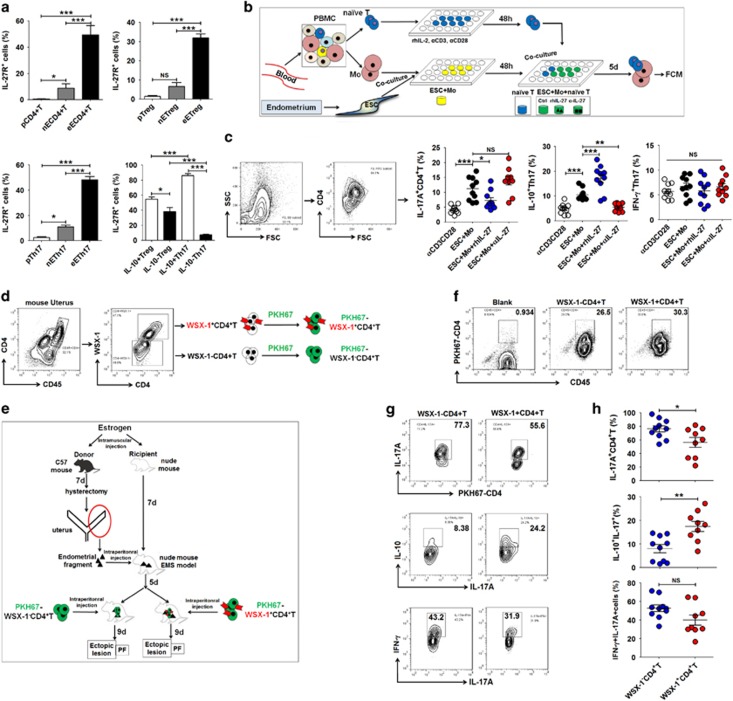
IL-27 induces IL-10 production of Th17 cells in the endometriotic milieu. (**a**) The percentage of IL-27 receptors (WSX-1 and gp130)^+^CD4^+^ T, Treg and Th17 cells from peripheral blood (*n*=6), normal endometrium (*n*=6) and ectopic lesion (*n*=5) by flow cytometry. The right panel is from an ectopic lesion (one-way ANOVA). (**b** and **c**) ESCs were co-cultured with monocytes from peripheral blood (*n*=10) for 48 h, and then naive T cells were added to the co-culture system and further treated with or without rhIL-27 (100 ng/ml) or anti-human IL-27 neutralizing antibody (*α*IL-27, 5 *μ*g/ml) for 5 days. Then, Th17 differentiation and IL-10 and IFN-*γ* levels in Th17 cells were detected by flow cytometry. Before co-culture, naive T cells were activated with anti-CD3 (5 *μ*g/ml), anti-CD28 (1 *μ*g/ml), and rhIL-2 (20 U/ml) for 2 days (one-way ANOVA). (**d** and **e**) We constructed an allotransplantation of intraperitoneal endometriosis model (*n*=10/group). On day 0, the uterus of female C57BL/6 mice was minced, and the tissue debris was intraperitoneally injected into nude mice. On day 5, the WSX-1^−^CD4^+^ T cells or WSX-1^+^CD4^+^ T cells from the uterus of female C57BL/6 mice collected by cell sorting were labeled with PKH-67 and transferred to the abdominal cavity in endometriosis nude mice. In addition, PBS was used as the control. On day 14, the endometriosis-like lesions and PF were collected and detected. (**f**) The characterization of transferred PKH-67-WSX-1^−^CD4^+^ T cells and PKH-67-WSX-1^+^CD4^+^ T cells in the PF from endometriosis mice by flow cytometry. (**g** and **h**) The Th17 differentiation, IL-10^+^Th17 and IFN-*γ*^+^Th17 cells in the PF from endometriosis mice by flow cytometry. WSX-1-CD4+T, transferred WSX-1^−^CD4^+^ T cells; WSX-1+CD4+T, transferred WSX-1^+^CD4^+^ T cells (Student's *t*-test). The data are expressed as the mean±S.E.M. *P<0.05, **P<0.01 and ***P<0.001

Of note, IL-27R is a heterodimer composed of the orphan cytokine receptor WSX-1 (also known as IL-27R*α*) and a signal-transducing chain glycoprotein 130 (gp130).^22^ Both of the receptor subunits are required for IL-27 signaling. IL-27R*α* is unique to IL-27R, whereas the gp130 subunit is shared with receptors for IL-6 and IL-35. As shown, peripheral WSX-1^+^gp130^+^ CD4^+^ T cells were extremely low while nearly half of CD4^+^ T cells in the endometrium from the ectopic lesion co-expressed WSX-1 and gp130 (Figure 4a and Supplementary Figures 6A–C). Among these, the IL-27R level on IL-10^+^Th17 cells from the ectopic lesion was significantly higher than that on IL-10^−^Th17 cells, and this difference was stronger than IL-10^+^ and IL-10^−^ Treg cells (Figure 4a and Supplementary Figure 6D).

To further investigate the role of IL-27 on Th17 differentiation and its IL-10 production, we stimulated naive T cells with recombinant IL-27 protein under Th17-polarizing conditions. Subsequently, we found that IL-27 restricted Th17 cell differentiation induced by IL-6 and TGF-*β*, whereas it significantly promoted IL-10, but not IFN-*γ*, production in Th17 cells. Co-cultured ESCs and monocytes significantly promoted both Th17 (Figures 4b, c, Supplementary Figure 7) and Treg (Supplementary Figure 8) cell differentiation. However, IL-27 induced high IL-10 production in Th17 (Figure 4c) but not Treg cells. Conversely, blocking IL-27 could eliminate the stimulatory effect of IL-10 production by Th17 cells medicated by co-culture of ESCs and monocytes (Figure 4c). To determine the role of IL-27/IL-27R signaling in IL-10 production in Th17 cells *in vivo*, we isolated WSX-1^+^ and WSX-1^−^ CD4^+^ T cells from C57 mouse uterus and transferred these PKH-67-labeled cells to the EMS nude mouse model by intraperitoneal injection (Figures 4d–f and Supplementary Figure 9). Consistent with the *in vitro* results, the mouse EMS model trials also provided evidence that IL-27/IL-27R signaling triggered an increase of IL-10^+^Th17 cells in the peritoneal flushing fluid along with a decrease of total Th17 cells (Figures 4g and h). Taken together, these results show that the expression of IL-27 and IL-27R has obvious specificity for tissues and cells. This characteristic creates a favorable condition for occurring IL-27-driving the IL-10^+^Th17 cell population in EMS.

### A c-Maf/ROR*γ*t/Blimp-1 signal is required for IL-10^+^Th17 cells triggered by IL-27

**Figure 5:**
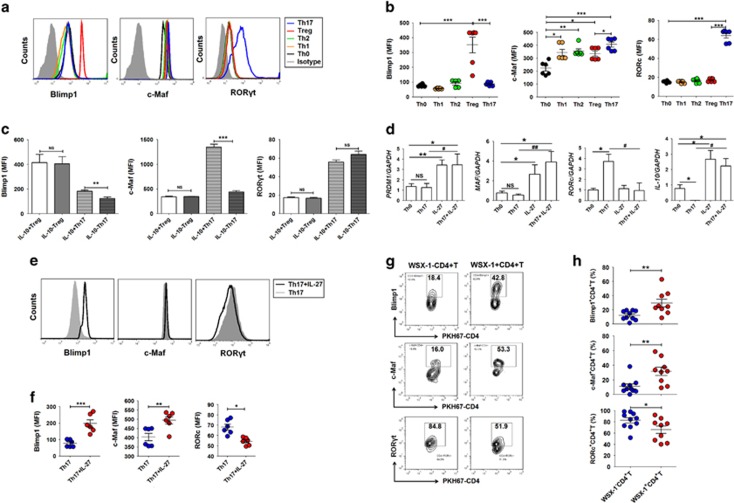
IL-27 induces high levels of C-Maf and Blimp-1 and a low level of ROR*γ*t in Th17 cells. (**a–c**) The mouse naive T cells (*n*=6) were differentiated to Th1 (stimulation with rhIL-2: 10 ng/ml; *α*CD3: 10 *μ*g/ml; *α*CD28: 2 *μ*g/ml; rhIFN-*γ*: 10 ng/ml; rhIL-12: 10 ng/ml; *α*IL-4: 10 *μ*g/ml), Th2 (rhIL-2: 10 ng/ml; *α*CD3: 10 *μ*g/ml; *α*CD28: 2 *μ*g/ml; IL-4: 10 ng/ml; *α*IFN-*γ*: 10 *μ*g/ml; *α*IL-12: 10 *μ*g/ml), Treg (rhIL-2: 10 ng/ml; *α*CD3: 10 *μ*g/ml; *α*CD28: 2 *μ*g/ml; rhTGF-*β*: 30 ng/ml) or Th17 (rhIL-2: 10 ng/ml; *α*CD3: 10 *μ*g/ml; *α*CD28: 2 *μ*g/ml; rhTGF-*β*: 2 ng/ml; rhIL-6: 50 ng/ml) cells for 5 days *in vitro*, and then the MFI of Blimp-1, c-Maf and ROR*γ*t in these cells was analyzed by flow cytometry (one-way ANOVA). (**d**) Human naive T cells (*n*=5) were differentiated into Th17 cells (rhIL-6: 50 ng/ml; rhTGF-*β*: 2 ng/ml; *α*CD3: 10 *μ*g/ml; *α*CD28: 2 *μ*g/ml; *α*IFN-*γ*: 10 *μ*g/ml; *α*IL-4: 10 *μ*g/ml; *α*IL-12: 10 *μ*g/ml) and stimulated with or without rhIL-27 (25 ng/ml) for 5 days. Then, the mRNA level of *PRDM1*, *MAF*, *RORc* and *IL-10* in Th17 cells was analyzed by real-time PCR. IL-27: rhIL-27 (one-way ANOVA). (**e** and **f**) Mouse naive T cells (*n*=6) were differentiated into Th17 cells and stimulated with or without rmIL-27 (25 ng/ml) *in vitro* for 5 days. Then, the MFI level of Blimp-1, c-Maf and ROR*γ*t in Th17 cells was analyzed by flow cytometry. IL-27: rmIL-27 (Student's *t*-test). (**g** and **h**) The level of Blimp-1, c-Maf and ROR*γ*t in CD4^+^ T cells (*n*=10) from the PF of mice by flow cytometry. The mouse endometriosis model was constructed according to the procedure of Figure 4d (Student's *t*-test). The data are expressed as the mean±S.E.M. *P<0.05, **P<0.01 and ***P<0.001; ^#^*P*<0.05, ^##^*P*<0.01 *versus* Th17 group

Owing to the important role of c-Maf and Blimp-1 in IL-27-induced IL-10 production,^23, 24, 25^ we next evaluated the expression of c-Maf, Blimp-1 and ROR*γ*t in different Th cell subsets. Among these, only c-Maf and ROR*γ*t were prominently expressed in Th17 cells. However, c-Maf and Blimp-1 were preferentially expressed in IL-10^+^Th17 cells compared with IL-10^−^Th17 cells (Figures 5a–c and Supplementary Figure 10). Under Th17-polarizing conditions, exposure to IL-27 promoted the transcription of *PRDM1*, *MAF* and *IL-10*, and inhibited *RORc* (Figure 5d). Meanwhile, the process occurred simultaneously with an elevation of Blimp-1 and c-Maf and a decrease of ROR*γ*t (Figures 5e and f). In the mouse model, transferring WSX-1^+^CD4^+^ T cells led to an increase of Blimp-1 and c-Maf and a decrease of ROR*γ*t in T cells (Figures 5g and h).

**Figure 6:**
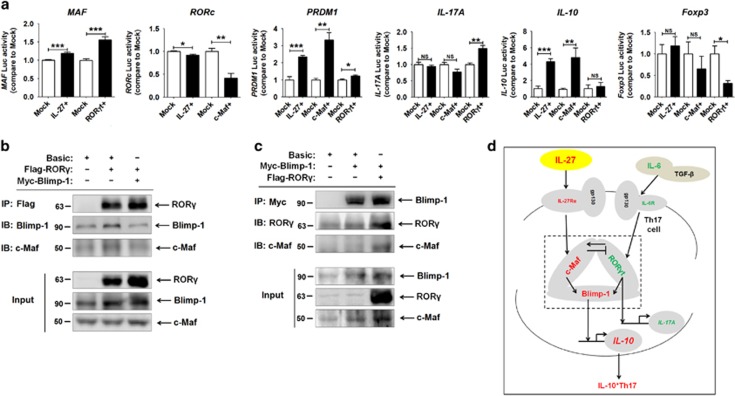
The c-Maf/ROR*γ*t/Blimp-1 complex is involved in IL-27-triggered IL-10^+^Th17 cells. (**a**) The relative luciferase activity of *MAF*, *RORc*, *PRDM1*, *IL-17A*, *IL-10* and *Foxp3* in HEK-293T cells was analyzed by a dual luciferase reporter assay, after co-transfection with overexpression plasmids (pIRES2-IL-27 plasmid, CMV-MCS-C-Maf-3Flag, CMV-MCS- ROR*γ*t-3Flag or mock plasmids) and luciferase reporter plasmids (PGL3-MAF-Luc, PGL3-RORc-Luc, PGL3-PRDM1-Luc, PGL3-IL-17A-Luc, PGL3-IL-10-Luc or PGL3-Foxp3-Luc). The data are expressed as the mean±S.E.M. *P<0.05, **P<0.01 and ***P<0.001 (Student's *t*-test). (**b** and **c**) The combination between Blimp-1, c-Maf and ROR*γ*t in HEK-293T cells was detected by co-IP assay. Flag-ROR*γ*, transfection with CMV-MCS-ROR*γ*t-3Flag plasmids; Myc-Blimp-1, transfection with Blimp-1-Myc plasmids. (**d**) The schematic role of IL-27 in IL-10^+^Th17 differentiation

Next, we utilized a dual luciferase reporter assay to identify the regulation relationship between c-Maf, Blimp-1 and ROR*γ*t (Supplementary Figure 11). Overexpression of IL-27 and c-Maf significantly transactivated *PRDM1* and *IL-10* levels and downregulated *RORc* levels but had no effect on *IL-17A* and *Foxp3* in HEK-293T cells (Figure 6a). However, ROR*γ*t overexpression led to a slight increase of *PRDM1* and an obvious elevation of *IL-17A* transcription and a decrease of *Foxp3* transcription (Figure 6a). There was no change in *IL-10* transcription after transfection with a ROR*γ*t-overexpressing plasmid.

*MAF* can bind to and transactivate the mouse IL-10 promoter in Th17 cells.^12^ In addition, Blimp-1 has an important role in IL-27-driven IL-10 production in Tr1 cells, which occurred in a c-Maf-dependent or independent manner.^14, 24, 25^ According to the prediction using the software SoftBerry, only the sequence of *PRDM1* had six loci for possible *IL-10* promoter region binding. These data described above indicate that c-Maf and ROR*γ*t may be the molecules upstream of Blimp-1. Under stimulation with IL-27, Blimp-1 could be involved in directly binding and activating *IL-10* transcription in human Th17 cells.

Further analysis by co-IP assay showed that c-Maf, Blimp-1 and ROR*γ*t could be combined together (Figures 6b and c). In addition, there was a positive regulation effect of ROR*γ*t on Blimp-1 (Figures 6b and c), which further echoed the results of a dual luciferase reporter assay. Collectively, these data suggest that the complex of c-Maf, Blimp-1 and ROR*γ*t may result in a significant upregulation of Blimp-1 and then lead to IL-10 production induced by IL-27 in Th17 cells (Figure 6d).

### IL-10^+^Th17 cells induced by IL-27 promoted the ectopic growth and implantation of ESC

**Figure 7:**
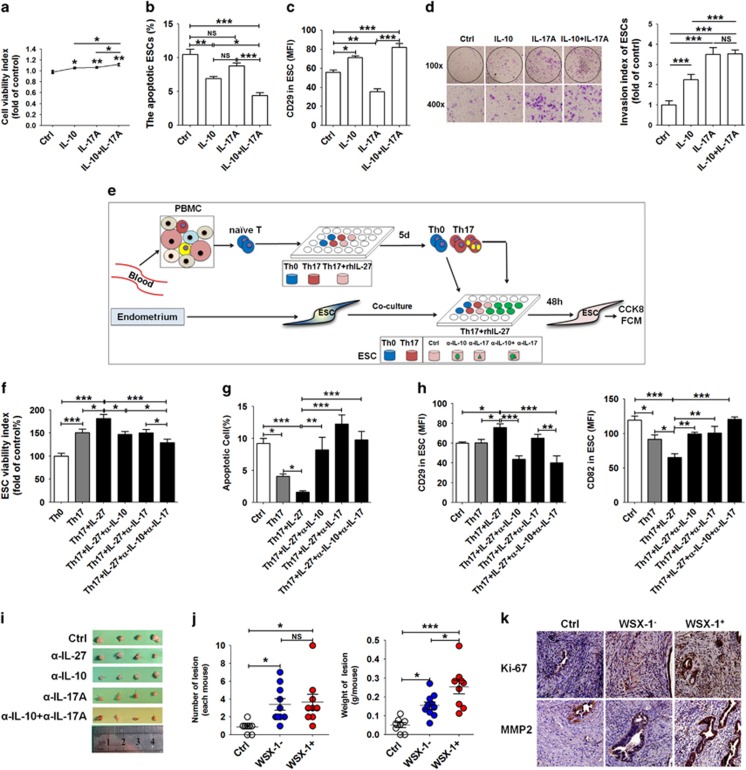
IL-10^+^Th17 cells induced by IL-27 promoted the ectopic growth and implantation of ESCs. (**a–d**) After treatment with rhIL-10 (100 ng/ml), rhIL-17A (10 ng/ml) or rhIL-10 plus rhIL-17A for 48 h, the viability (**a**), apoptosis (**b**), CD29 level (**c**) and invasiveness (**d**) of ESCs (*n*=5) were evaluated by CCK8 assay, apoptosis assay, flow cytometry and Matrigel invasion assay, respectively. Original magnification: × 200 (one-way ANOVA). (**e**–**h**) The human naive T cells were polarized to Th17 *in vitro*, and stimulated with or without rhIL-27. Then, these cells were collected and indirectly co-cultured with ESCs for 48 h, and *α*IL-10 and or *α*IL-17 were added to the co-culture system (**e**). Then, the viability (**f**), apoptosis (**g**) and the MFI of CD29 and CD82 (**h**) of ESCs (*n*=5) were evaluated by CCK8 assay, apoptosis assay and flow cytometry, respectively (one-way ANOVA). (**i**) The size of endometriosis-like lesions from the C57BL/6 endometriosis mouse model was measured after treatment with *α*IL-27 (50 *μ*g/mouse), *α*IL-10 (50 *μ*g/mouse) and/or *α*IL-17A (50 *μ*g/mouse). (**j**) The number and weight of endometriosis-like lesions from the nude mouse endometriosis model was measured after transferring WSX-1^−^CD4^+^ T (*n*=10) or WSX-1^+^CD4^+^ T (*n*=9) cells. Ctrl, PBS treatment; WSX−, transferred WSX-1^−^CD4^+^ T cells; WSX+, transferred WSX-1^+^CD4^+^ T cells (one-way ANOVA). (**k**) The expression of Ki-67 and MMP2 in endometriosis-like lesions from the nude mouse endometriosis model was analyzed by flow cytometry (one-way ANOVA). Original magnification: × 200. The data are expressed as the mean±S.E.M. *P<0.05, **P<0.01 and ***P<0.001

Compared with normal cells, ectopic ESCs have higher greater viability and invasiveness and a lower apoptotic level (Supplementary Figure 12). IL-17A stimulation *in vitro* promoted the viability and invasion, but repressed the ESC apoptosis and adhesion to the extracellular matrix (ECM) such as fibronectin and collagen I, especially ectopic ESC (Supplementary Figure 12). A combination of IL-10 with IL-17A further enhanced effects on viability, apoptosis, but not invasion, of ESCs (Figures 7a–d). In contrast, IL-10 could reverse the inhibitory effect on adhesion-related molecule CD29 (also named integrin*β*1)^26^ mediated by IL-17A (Figure 7c). These results indicate that IL-17A mainly promotes proliferation and invasion, and restricts the adhesion of ESCs, thereby accelerating the growth, implantation and dissemination of an ectopic lesion during the initial stage of disease. With the progression of EMS, increase of IL-10 levels in the endometriotic milieu will further stimulate growth, adhesion and deep infiltration of the ectopic lesion.

Next, we sought to investigate the role of these IL-10-producing Th17 cells in the regulation of ESC biological behaviors (Figure 7e). IL-27 significantly strengthened the effect of polarized Th17 cells on viability, apoptosis and expression of CD29 and metastasis suppressor protein CD82 *in vitro*^27^ (Figures 7f–h). However, blockade of IL-10 and IL-17 could effectively reverse the effects induced by Th17 cells plus IL-27 treatment (Figures 7f–h). The decrease of ectopic lesion size occurred in IL-27, IL-10 and/or IL-17-blocked mice, especially in the IL-10 plus IL-17 blockade (Figure 7i and Supplementary Figure 13). Further analysis of the role of IL-27 signaling in the development of EMS showed that transferring either WSX-1^+^ or WSX-1^−^ CD4^+^ T cells from a C57 mouse uterus led to a marked increase of ectopic lesion numbers (Figure 7j). Interestingly, WSX-1^+^CD4^+^ T-cell-transferred mice had the highest ectopic lesion weight (Figure 7j), and higher levels of Ki-67 and matrix metallopeptidase 9 (MMP-9; Figure 7k).

Taken together, these data indicate that IL-10^+^Th17 cells induced by IL-27 obviously promote the growth and implantation of ectopic lesions and further accelerate the progression of the disease by functional molecules IL-17A and IL-10.

## Discussion

The plasticity of Th17 cells is reflected in their heterogeneity and inherent phenotypic instability,^28^ which is likely to be dependent on the local microenvironment. In Th17-cell-related diseases, the effective factors such as members of the IL-17 family can induce the release of pro-inflammatory and neutrophil-activated cytokines, activate DCs or macrophages, and promote tissue inflammation.^29^ IL-23 signaling is particularly important for the emergence of IL-17A^+^IFN-*γ*^+^ T cells, which are referred to as ‘Th17+Th1' cells in intestinal inflammation.^30^ Interestingly, Th17 cells have recently been shown to produce IL-10.^31, 32^ Although the fraction of IL-17A-producing cells within the IL-10-producing cell population was relatively small, this fraction represented up to 30% of all IL-17A producing T cells.^33^ With respect to antigen-specific Th17 cells, IL-10^+^Th17 cells are considered to have a regulatory phenotype. IL-17A-producing CD4^+^ T cells express IL-10 receptor *α* (IL-10R*α*).^33^ IL-10 produced by Th17 cells exerts suppressive effects to inhibit fully differentiated pathogenic Th17 populations, and the development of inflammation. However, induction of IL-10-producing Th17 cells is not well understood.

EMS is considered an inflammatory disease. In recent years, several reports showed that anti-inflammatory factors IL-4 and IL-10 were also increased in EMS.^3, 5, 6, 7, 8^ However, the lack of knowledge on the cytokine change rule from the perspective of disease progression, and unknown mechanisms for the formation and role of anti-inflammatory factors in local environment of EMS, leads to less awareness and acceptance of the importance of anti-inflammatory factors in EMS. Here, we evaluated the cytokine profile and the level and phenotype of Th17 cells in the PF of women with endometriosis, and observed that the PF of patients with endometriosis had high levels of pro-inflammatory cytokines, such as IL-17A, IFN-*γ*, TNF-*α*, IL-1*β* and Th17 cells, and cytokines for Th17 differentiation (IL-6 and TGF-*β*). However, anti-inflammatory cytokines, such as IL-10 and IL-4, and IL-10-producing Th17 cells were mainly elevated in advanced EMS. The results suggested that the local microenvironment of women with EMS presents a coexistent state of pro-inflammatory and tolerance factors. In the initial stage of this disease, the dominant position is pro-inflammatory. However, the environment tends towards tolerance during the advanced stage.

IL-27 is mainly secreted by APC following stimulation by microbial products or other immune stimuli. However, the regulatory factors for IL-27 expression are largely unclear. Here, we observed that the interaction between ESCs and macrophages in ectopic lesions led to a high level of IL-27, IL-6 and TGF-*β*. The accumulated IL-27 from ectopic lesions reflects the tissue and cell specificities of IL-27 expression. Co-culture with ESCs upregulated IL-27 expression in macrophages, especially co-culture with IL-27^over^ESCs, suggesting that IL-27 can further promote its secretion with positive feedback. Contrary to TGF-*β* alone, the combination of IL-6 and TGF-*β* resulted in an increase of IL-27 production by macrophages. Taking into account gp130 receptor shared by IL-6 and IL-27, the stimulatory effect of IL-6 on IL-27 and the self-enhancement of IL-27 may require gp130 signaling. Moreover, exposure to estrogen or TCDD also increased IL-27 levels in macrophages. These results underline the fact that high levels from endometrial macrophages and ESCs from patients with endometriosis might be contributed to by internal (local high level of estrogen, IL-6 and TGF-*β*) and external environment (TCDD exposure) factors.

IL-27 restricted Th17 cells, which were induced by a co-culture system (ESC-macrophage-naive T cell) *in vitro*, differentiated into a regulatory phenotype characteristic with a signature transcriptional profile and IL-10 production. These results were further supported by experiments *in vivo*. Previous studies reported that IL-27 had a dual effect on Treg differentiation.^34, 35, 36, 37^ However, IL-27 in the co-culture model of E-M-T did not influence Treg cell differentiation, possibly due to the special immune microenvironment of endometriotic lesions and cross-regulatory effects between IL-27 and TGF-*β*.^14^ In addition, TCDD stimulation promotes Treg cell differentiation and inhibits Th17 cell differentiation via aryl hydrocarbon receptor (Ahr).^38, 39^ Therefore, our data indicated a regulatory effect of TCDD/Ahr on Th17 cells but Treg cells should be dependent on IL-27.

Transcription factor Maf, also known as proto-oncogene c-Maf or V-maf musculoaponeurotic fibrosarcoma oncogene homolog is a transcription factor that is encoded by the *MAF* gene in humans.^40^ Under IL-27 stimulation, the activation and cooperation between Ahr and STAT3 activate IL-10 production in Tr1 cells.^22^ Moreover, IL-27 induces early growth response gene-2 (Egr-2), which is a transcriptional regulator for Blimp-1, which has an important role in IL-10 induction.^25^ Both STAT1 and STAT3 are involved in the above processes. Here, we have established that IL-10^+^Th17 cells are c-Maf^high^Blimp-1^high^ROR^high^. In advanced endometriosis, the formation of a c-Maf, ROR*γ*t and Blimp-1 complex triggered by IL-27 contributes to the expansion of IL-10-producing Th17 cells. As shown in Supplementary Figure 14, based on our results, previous reports, and the analysis of Pathway Commons Project, KEGG, Pathway Interaction Database and IntAct Database, we concluded that under the stimulation of IL-6 and TGF-*β*, STAT3 may be activated in naive T cells, which further promotes *RORc* and *IL-17A* transcription, and induces IL-17A production. If IL-27 co-exists in this environment, the interaction of IL-27 and receptors may activate AP-1 family transcription factors (such as JUN and JUNB), which further induce *PRDM1* and downstream *IL-10* transcription by inhibiting *RORc* and promoting *MAF*, finally triggering IL-10 production of Th17 cells.

As one of the key functional molecules for IL-10^+^Th17 cells, IL-17A mainly promotes proliferation and invasion, and restricts the adhesion of ESCs, thereby accelerating the growth, implantation and dissemination of ectopic lesions *in vitro* and *in vivo*. IL-17A also promotes angiogenesis and a pro-inflammatory environment in the peritoneal cavity for the establishment and maintenance of endometriosis lesions.^4^ With the cooperation of IL-10, there were obvious increases of growth, adhesion and deep infiltration of ectopic lesion. In addition to CD82 and integrin*β*1, IL-8, cyclooxygensase-2 (Cox-2) and MMPs may be involved in this process.^7, 41^ IL-27 further amplified the stimulatory effect of Th17 on the growth and invasion of ESCs *in vitro*, and increased the number and weight of ectopic lesions in the mouse endometriosis model.

**Figure 8:**
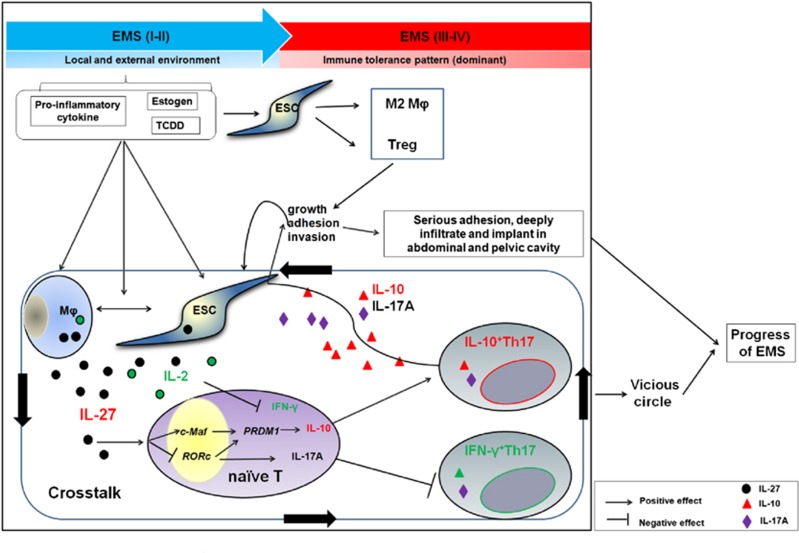
The schematic roles of IL-27 in the progression of endometriosis by inducing IL-10^+^Th17 differentiation. In the early stage of endometriosis, cytokines (such as IL-6 and TGF-*β*), high estrogen and TCDD exposure significantly promote macrophages in the local microenvironment of ectopic foci to secrete high levels of IL-27. Owing to the interaction between macrophages and ESCs in ectopic lesions, there is an accumulation of IL-27 in the microenvironment of ectopic foci. IL-27 inhibits Th17 differentiation, and promotes the production of IL-10 in Th17 cells via the c-Maf/RORC/Blimp-1 complex, participating in the formation of an immune tolerance pattern in the late stage of endometriosis. These IL-10-produced Th17 cells promote the growth, adhesion, invasion and deep infiltration of ESCs, thus accelerating the progression of endometriosis. With the progression of this disease, the growth of ectopic ESCs may result in increased recruitment and proliferation of macrophages and IL-27 levels in the microenvironment of ectopic foci. These changes will form a vicious circle and accelerate the development of endometriosis

Recent research has reported that an estrogen receptor agonist also induces IL-10 production in Th17 cells.^42^ As an estrogen-dependent inflammatory disease (Figure 8), in the initiation stage of endometriosis, cytokines, high estrogen and TCDD exposure significantly promote macrophages in the local microenvironment of ectopic foci to secrete a high level of IL-27. Owing to the interaction between macrophages and ESCs in ectopic lesions, there is an accumulation of IL-27 in the microenvironment of ectopic foci. IL-27 inhibits Th17 differentiation, and promotes the production of IL-10 in Th17 cells by the c-Maf/RORC/Blimp-1 complex, participating in the formation of an immune tolerance pattern in the late stage of endometriosis. These IL-10 and IL-17A double-producing Th17 cells promote the growth, adhesion, invasion and deep infiltration of ESCs, thus accelerating the progression of endometriosis. With the progression of this disease, the growth of ectopic ESCs may result in increased recruitment and proliferation of macrophages and IL-27 levels in the microenvironment of ectopic foci. These changes will form a vicious circle in a positive feedback loop and finally accelerate the development of endometriosis.

## Materials and Methods

### Patients, laparoscopies and tissues collection

The protocol for this study was approved by the Human Research Ethics Committee of Obstetrics and Gynecology Hospital, Fudan University (2012-4-11), and written informed consent was obtained from all of the participants. Laparoscopic surgeries were performed in reproductive age women (mean age 37.4 years; range 30–43) at the Obstetrics and Gynecology Hospital of Fudan University. Clinical suspicion of endometriosis was based on patient symptoms and included dysmenorrhea, deep dyspareunia, chronic pelvic pain, infertility and cyclical alterations in bowel and urinary habits occurring only during menstruation. After physical examination, the patients were subjected to transvaginal ultrasound or MRI. According to the suspicion of the presence of deep infiltrating lesions or if the patient had persistent pain or infertility, a surgical laparoscopic procedure was indicated. During laparoscopy, 2–10 ml of peritoneal fluid was collected from the anterior and/or posterior cul-de-sac, and biopsies from deep infiltrating lesions were obtained from each patient. Based on histopathology and medical records, patients with superficial peritoneal endometriosis (SPE), ovarian endometrioma (OMA), adenomyosis or pelvic inflammatory disease (PID) related infertility were excluded. Finally, a total of 58 patients with endometriosis were grouped into rAFS stage I–II (*n*=24) and rAFS stage III–IV (*n*=34), according to the revised system of the American Society of Reproductive Medicine, 1996 (American Society for Reproductive Medicine, 1997). Normal endometrium was obtained at hysterectomy from patients with uterine leiomyoma (*n*=83) but without endometriosis and/or adenomyosis as healthy controls. None of the included patients had experienced complications related to pelvic inflammatory disease and none took any medications or received hormonal therapy within 6 months before surgery. All the samples were obtained in the proliferative phase of the cycle, which was confirmed histologically according to established criteria.

### Collection and preparation of PF and cell culture

Detailed information about PF collection and cell culture was provided in the Supplementary Information.

### Immunohistochemistry

Detailed information about immunohistochemistry analysis was provided in the Supplementary Information.

### Isolation and purity of immune cells

PBMCs were isolated from healthy fertile women. Naive CD4^+^ T cells and CD14^+^ monocytes/macrophages were isolated from PBMCs using magnetic beads (Miltenyi Biotec, Bergisch Gladbach, Germany) for use in subsequent *in vitro* experiments. The WSX-1^+^ and WSX^−^ CD4^+^ T cells were sorted from mouse uterus by fluorescence-activated cell sorting (FACS), and then these cells were labeled with PKH-67 (green fluorescence dye, Sigma-Aldrich Co., St. Louis, MO, USA).

### Enzyme-linked immunosorbent assay

The levels of cytokines IL-6 and TGF-*β* were detected and analyzed by Bio-Plex Suspension Array (Bio-Rad Laboratories, Inc., Hercules, CA, USA). The secretion level of IL-27 was detected by the Human IL-27 ELISA Kit (Cat. No. 434607, LEGEND MAX, BioLegend, San Diego, CA, USA).

### Dual luciferase reporter assay

The overexpression and luciferase reporter plasmids were constructed by GeneChem Co., Ltd (Shanghai, China). These plasmids were transfected into HEK-293T cells by Effectene Transfection Reagent (Qiagen, Hilden, Germany) as described in the Supplementary Information. The relative luciferase activity was analyzed by the Dual Luciferase Reporter (DLR) Assay (Promega, Madison, WI, USA).

### The transcription of *PRDM1*, *MAF*, *RORc* and *IL-10*

Total RNA was extracted from Th17 cells using an RNeasy Mini kit according to the manufacturer's protocol (Qiagen). Then the transcription level of *PRDM1, MAF*, *RORc* and *IL-10* in CD4^+^ T cells was analyzed by real-time PCR. Real-time PCR was performed using an ABI PRISMTM 7900 Sequence Detector (Applied Biosystems, Warrington, UK). The primer sequences were designed and synthesized by TaKaRa Biotechnology Co., Ltd (Tokyo, Japan) as described in the Supplementary Information. The expression levels of the samples were expressed as arbitrary units defined by the 2^−ΔΔCT^ method. All the measurements were performed in triplicate. The specificity of the product was assessed by melting curve analysis.

### Immunoprecipitation and immunoblotting

The HEK-293T cells (2 × 10^6^ cells/well) were transected with ROR*γ*-Flag (5 *μ*g) and or Blimp-1-Myc (5 *μ*g) plasmids. According to the standard procedure, immunoprecipitation and immunoblotting were performed to analyze the combination of c-Maf (1:500, sc-7866, Santa Cruz, Dallas, TX, USA), Blimp-1 (1:500, sc-47732, Santa Cruz) and ROR*γ* (1:500 sc-81371, Santa Cruz).

### Flow cytometry

To identify and evaluate the Th17 cells, the mononuclear cells from peritoneal fluid were stained with anti-CD45 Ab and anti-CD4 Ab, followed by intracellular staining of IL-17A plus IL-10 and IFN-*γ* according to the manufacturer's instructions. In addition, flow cytometry was performed to analyze the percentage of Th17 cells, IL-10 and IFN-*γ* levels, WSX-1, gp130, Blimp-1, c-Maf and ROR*γ*t in CD4^+^ T cells, the expression of IL-27 in monocytes/macrophages, and the IL-27, CD28 and CD29 levels in ESCs, using isotypic IgG antibodies as controls. The samples were analyzed using a FACS Calibur flow cytometer (Becton Dickinson, Franklin Lakes, NJ, USA) and Cellquest software (Becton Dickinson). The level of cytokines in PF was detected by CBA assay (BD, San Jose, CA, USA).

### Cell viability, apoptosis and matrigel invasion assays

Detailed information for cell viability, apoptosis and Matrigel invasion assays was provided in the Supplementary Information.

### Intraperitoneal endometriosis model

A group of adult female C57BL/6 mice and nude mice were purchased from the Laboratory Animal Facility of Fudan University and used for this study. They were maintained for 2 weeks in the animal facility before use. The Animal Care and Use Committee of Obstetrics and Gynecology Hospital, Fudan University approved all of the animal protocols.

For the C57BL/6 mice, intraperitoneal endometriosis-like lesions were induced surgically by suturing uterine tissue samples to the abdominal wall. For the nude mice, we constructed an allotransplantation of intraperitoneal endometriosis model. The detailed information for the model was supplied in the Supplementary Information.

### Statistics

The continuous variables were shown as the mean±S.E.M. Continuous variables were analyzed by Student's *t*-test for two groups and by one-way ANOVA using Tukey's *post hoc* test in multiple groups. All the analyses were conducted by SPSS 16.0 Statistical Package for the Social Sciences software (IBM SPSS, Armonk, NY, USA). Statistical significance was considered at *P*<0.05.
