# Supplementary figures and images for: IL-27 triggers IL-10 production in Th17 cells via a c-Maf/RORγt/Blimp-1 signal to promote the progression of endometriosis
Source: Cell Death Dis. 2017 Mar 16;8(3):e2666–. doi: 10.1038/cddis.2017.95 (PMC5386585; doi:10.1038/cddis.2017.95)

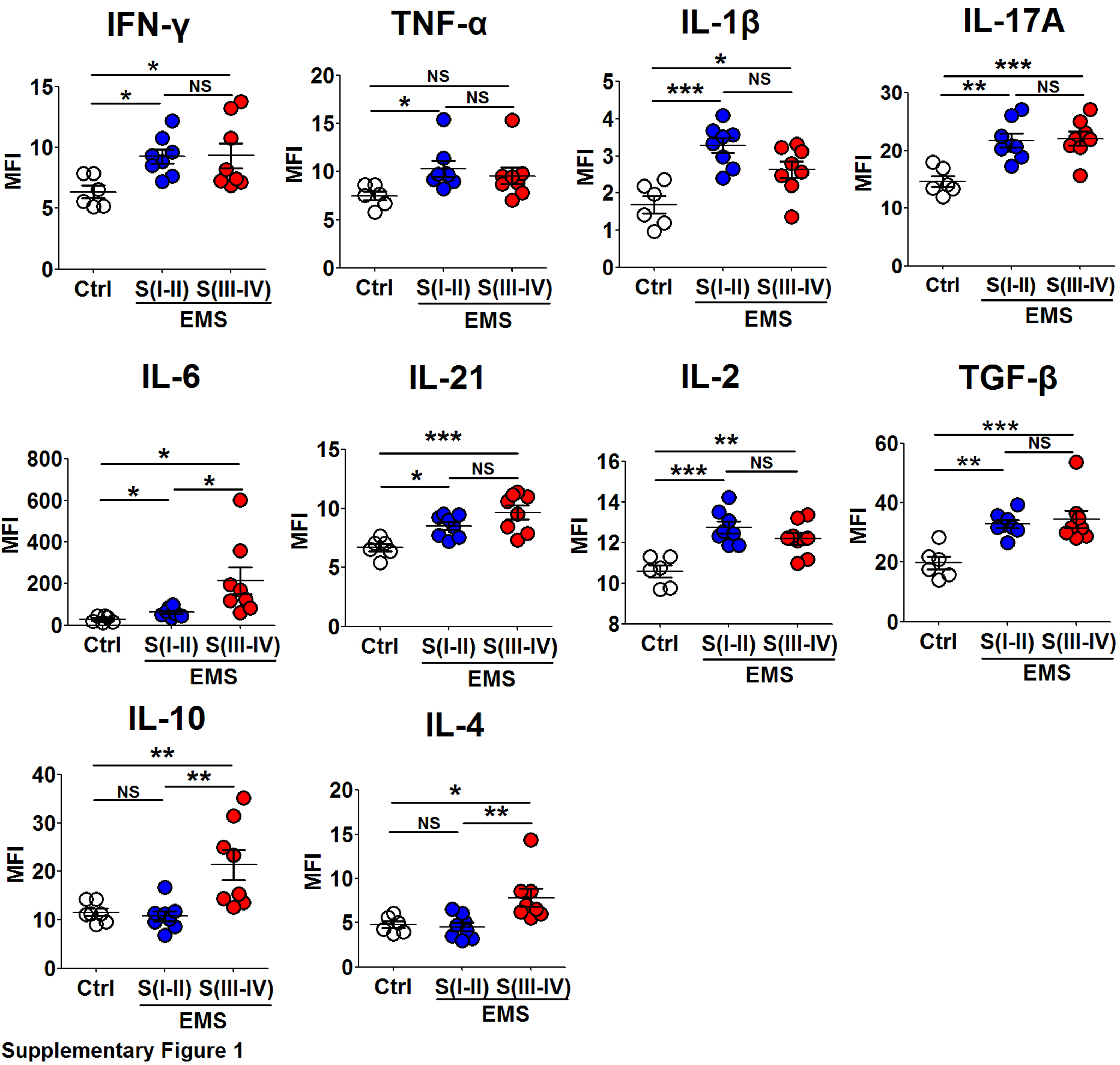

Supplement: Supplementary Figure 1 [file cddis201795x1.tif]

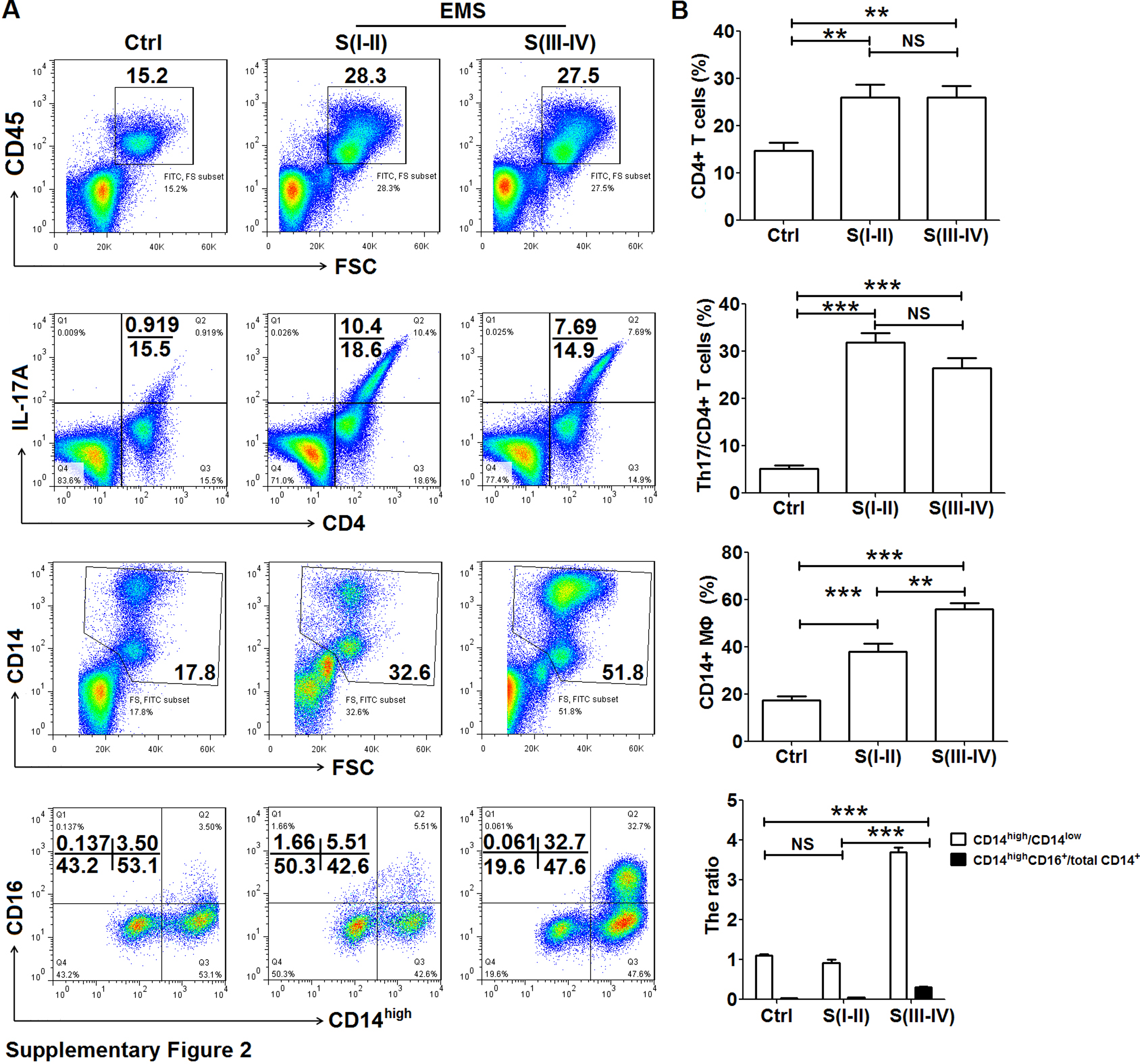

Supplement: Supplementary Figure 2 [file cddis201795x2.tif]

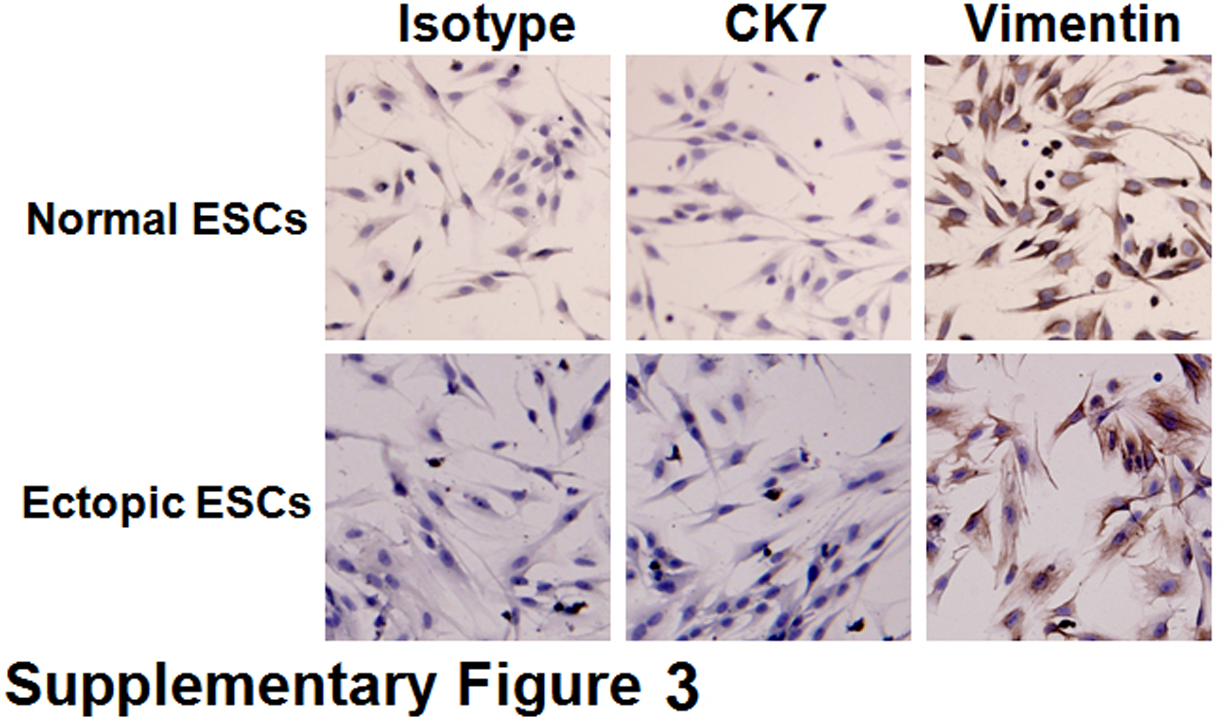

Supplement: Supplementary Figure 3 [file cddis201795x3.tif]

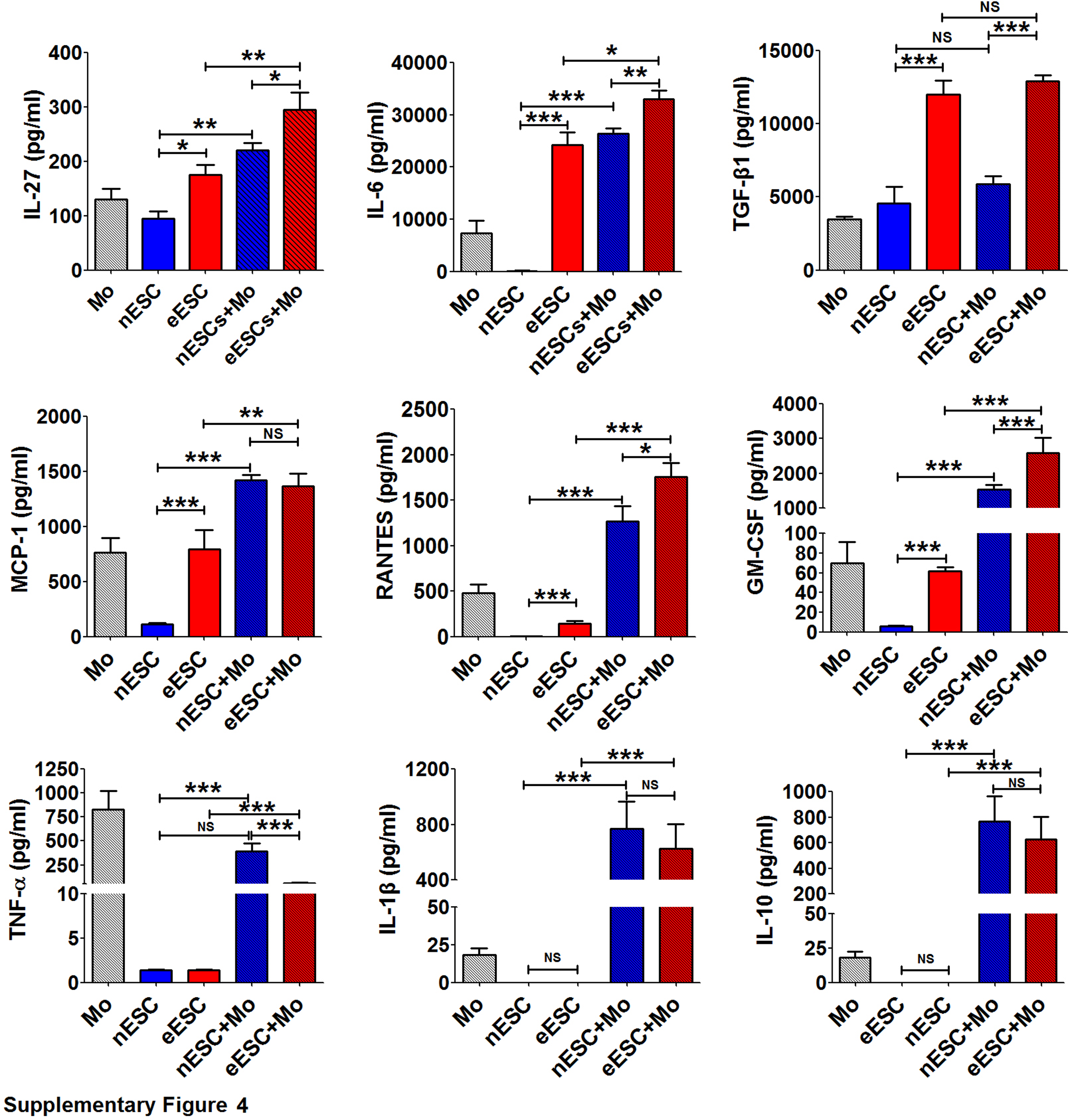

Supplement: Supplementary Figure 4 [file cddis201795x4.tif]

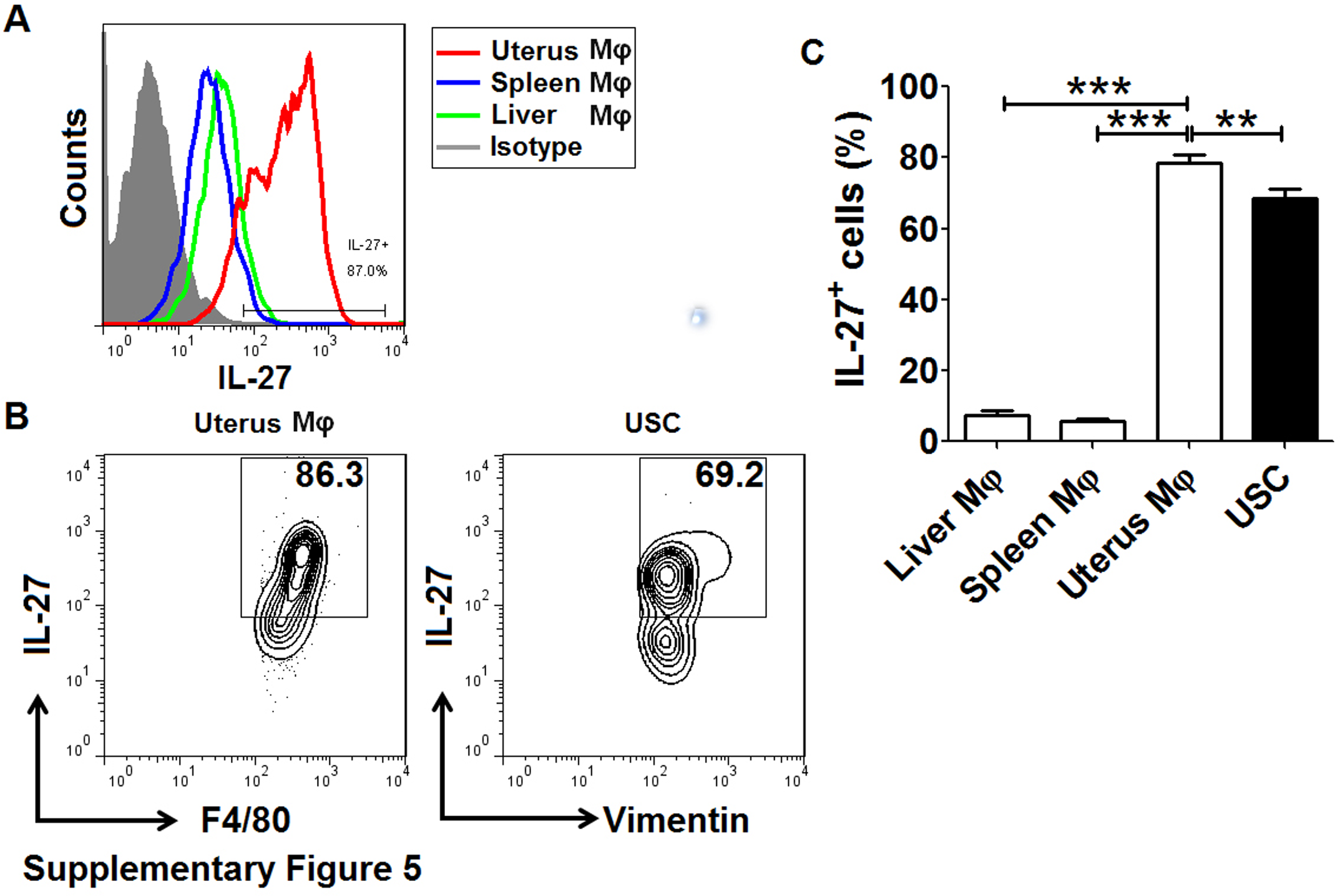

Supplement: Supplementary Figure 5 [file cddis201795x5.tif]

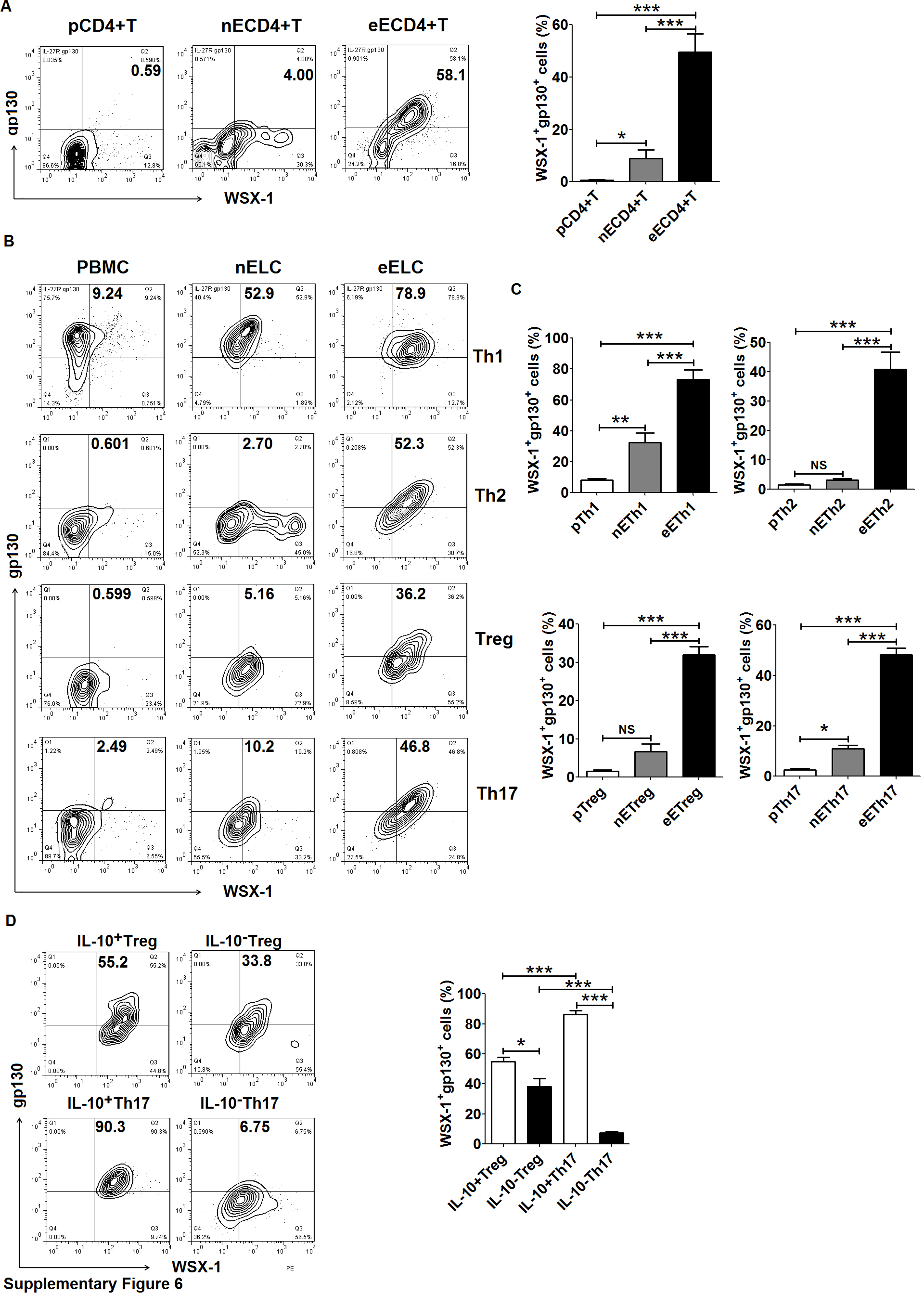

Supplement: Supplementary Figure 6 [file cddis201795x6.tif]

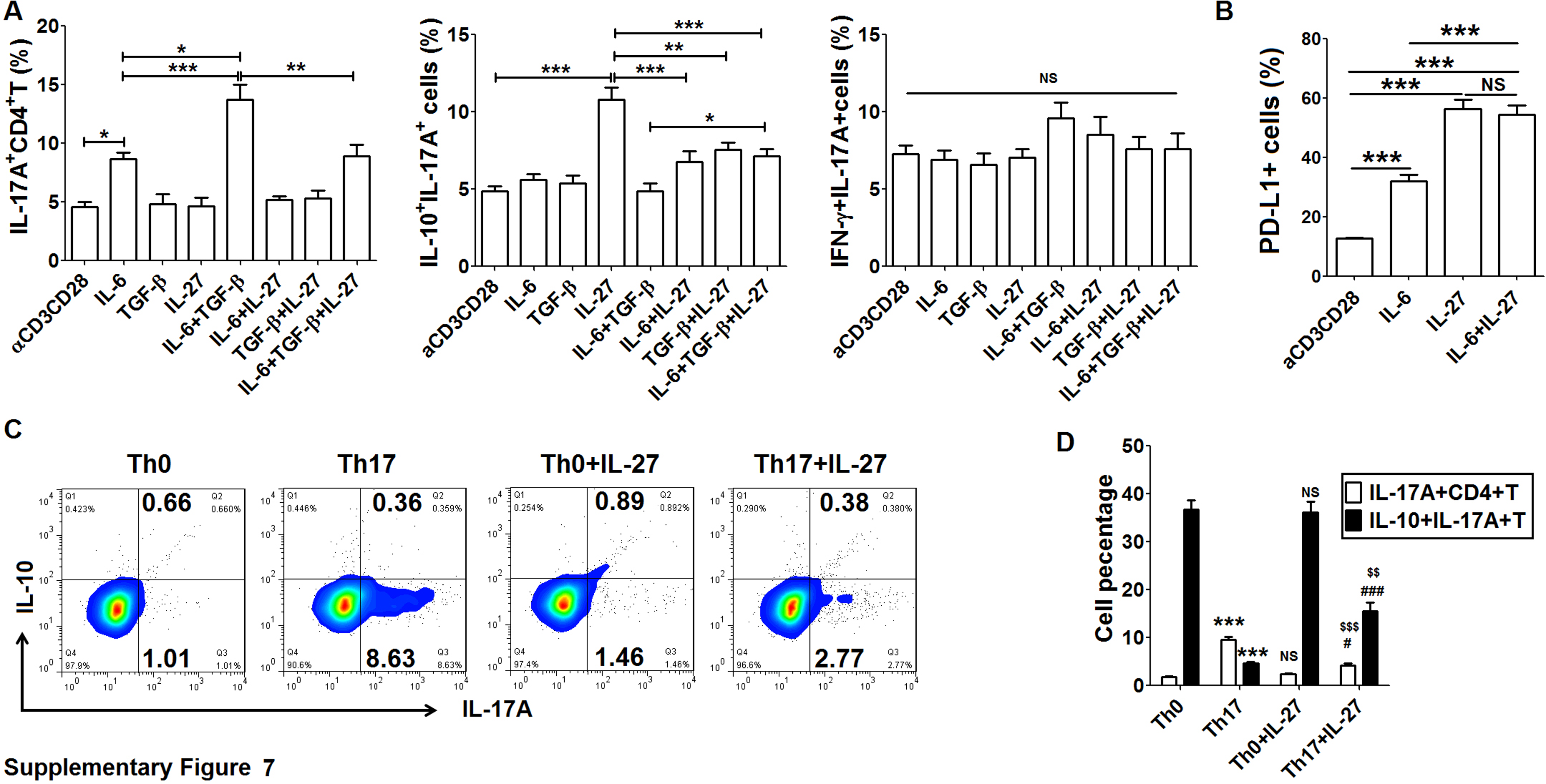

Supplement: Supplementary Figure 7 [file cddis201795x7.tif]

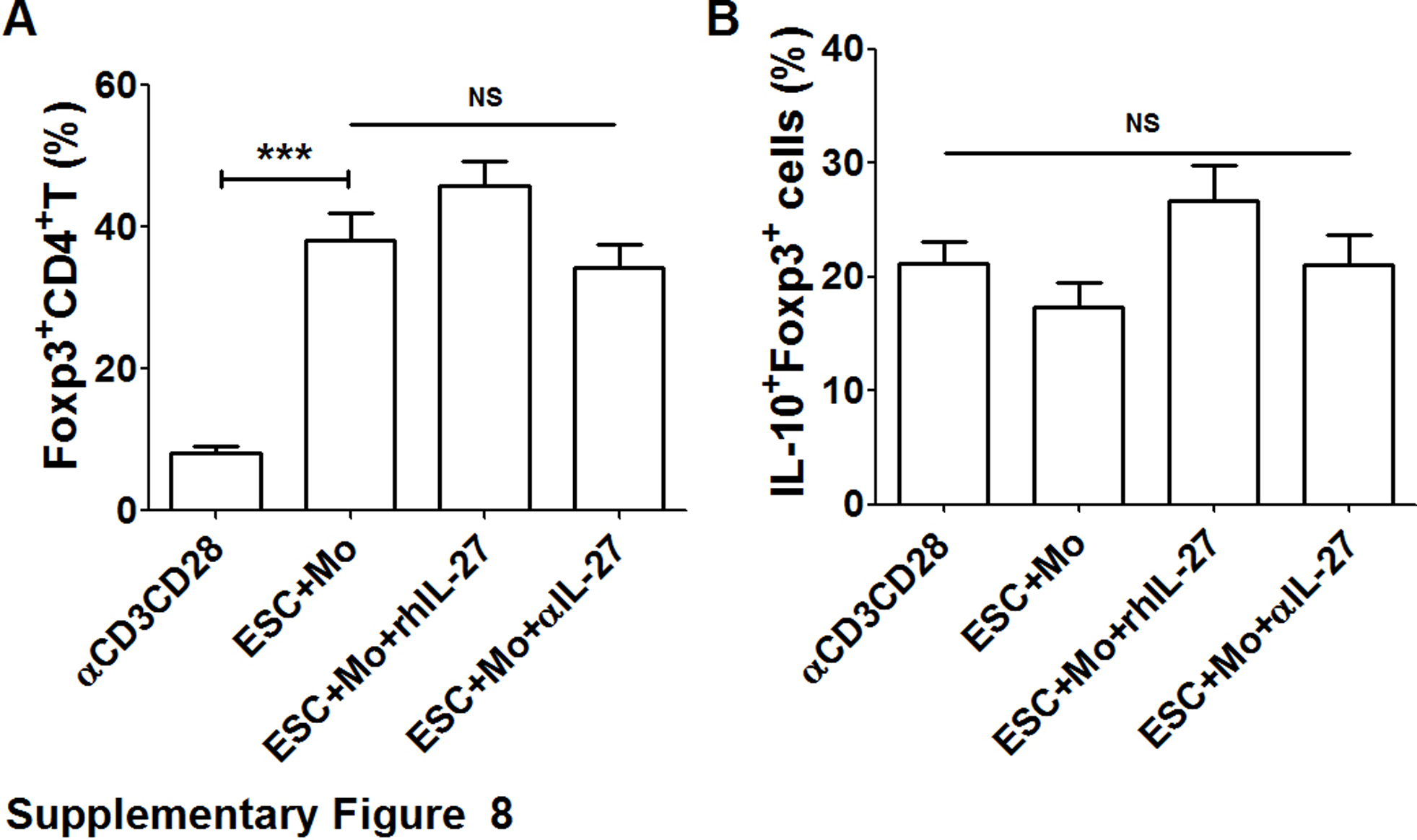

Supplement: Supplementary Figure 8 [file cddis201795x8.tif]

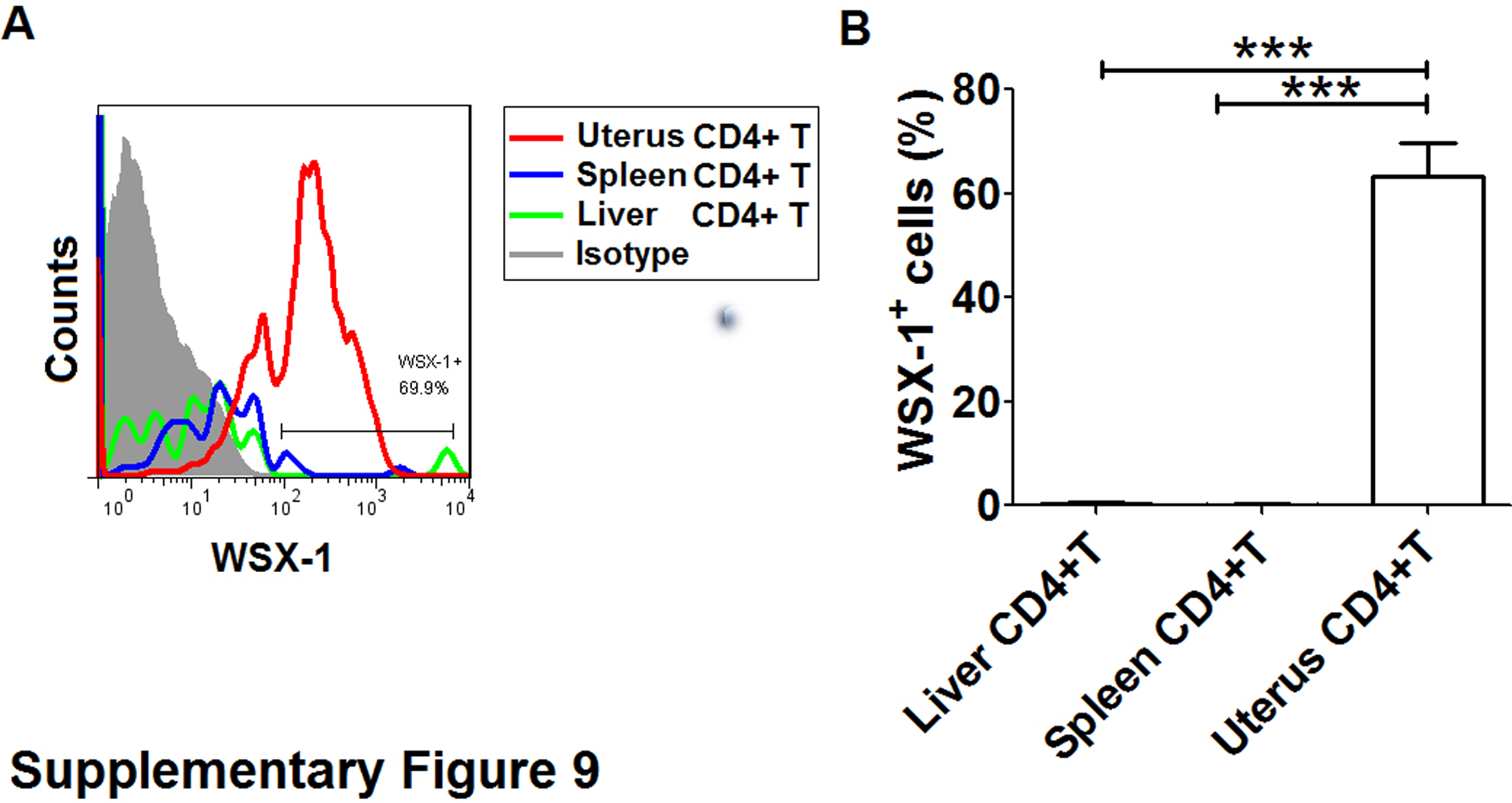

Supplement: Supplementary Figure 9 [file cddis201795x9.tif]

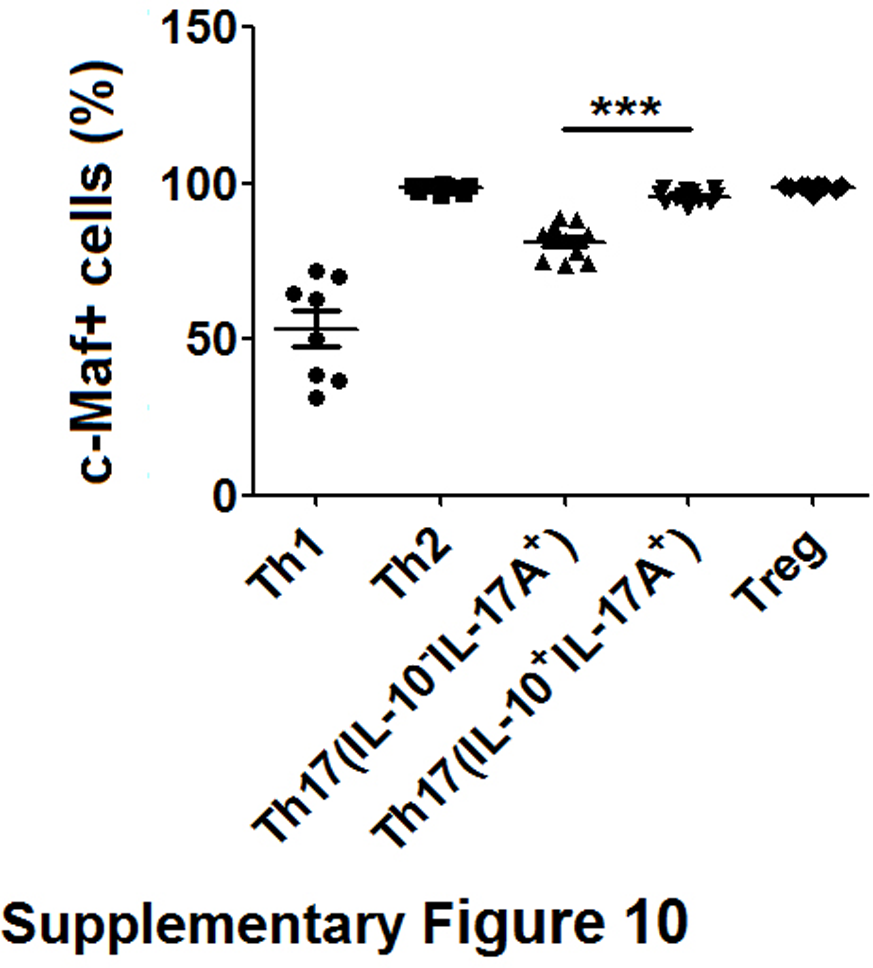

Supplement: Supplementary Figure 10 [file cddis201795x10.tif]

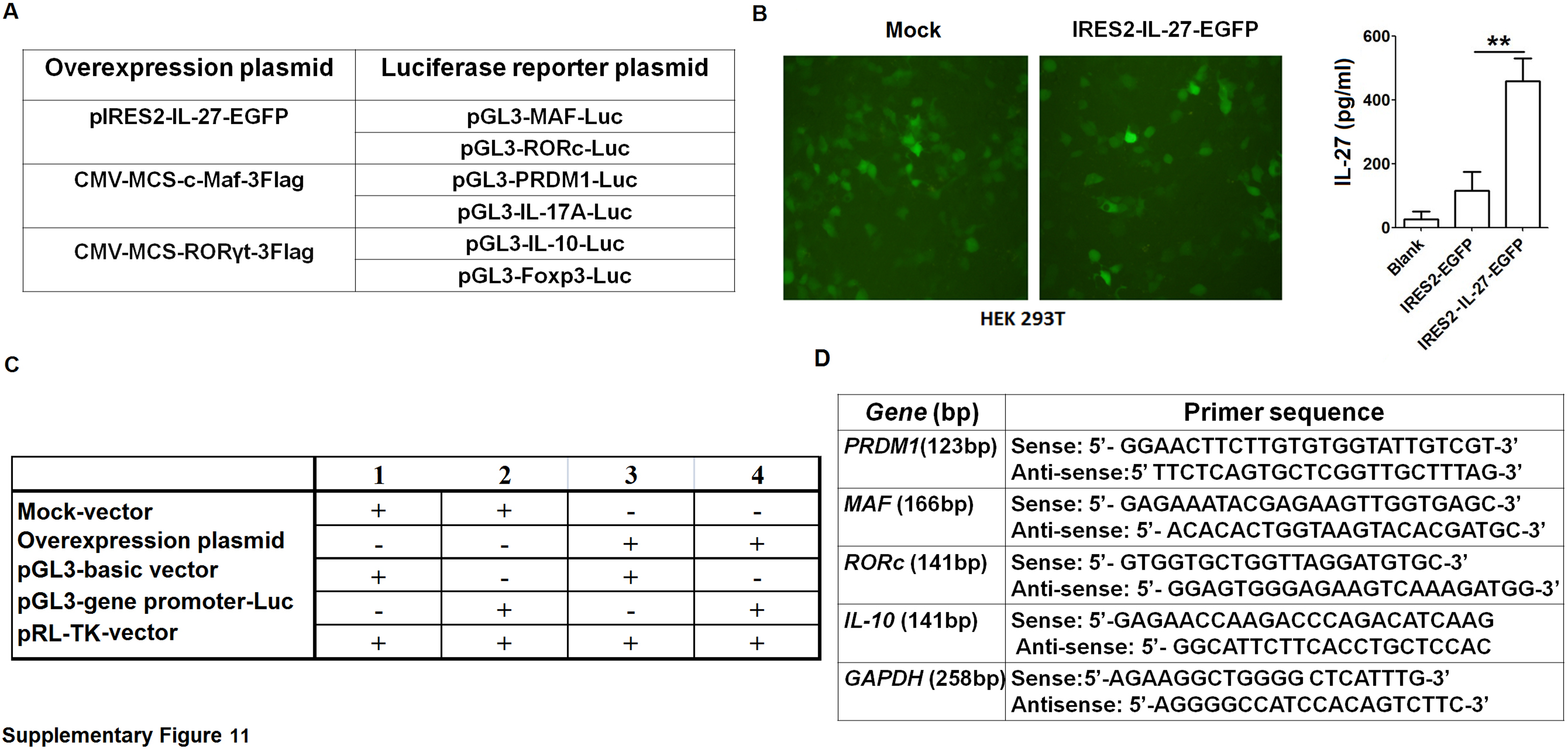

Supplement: Supplementary Figure 11 [file cddis201795x11.tif]

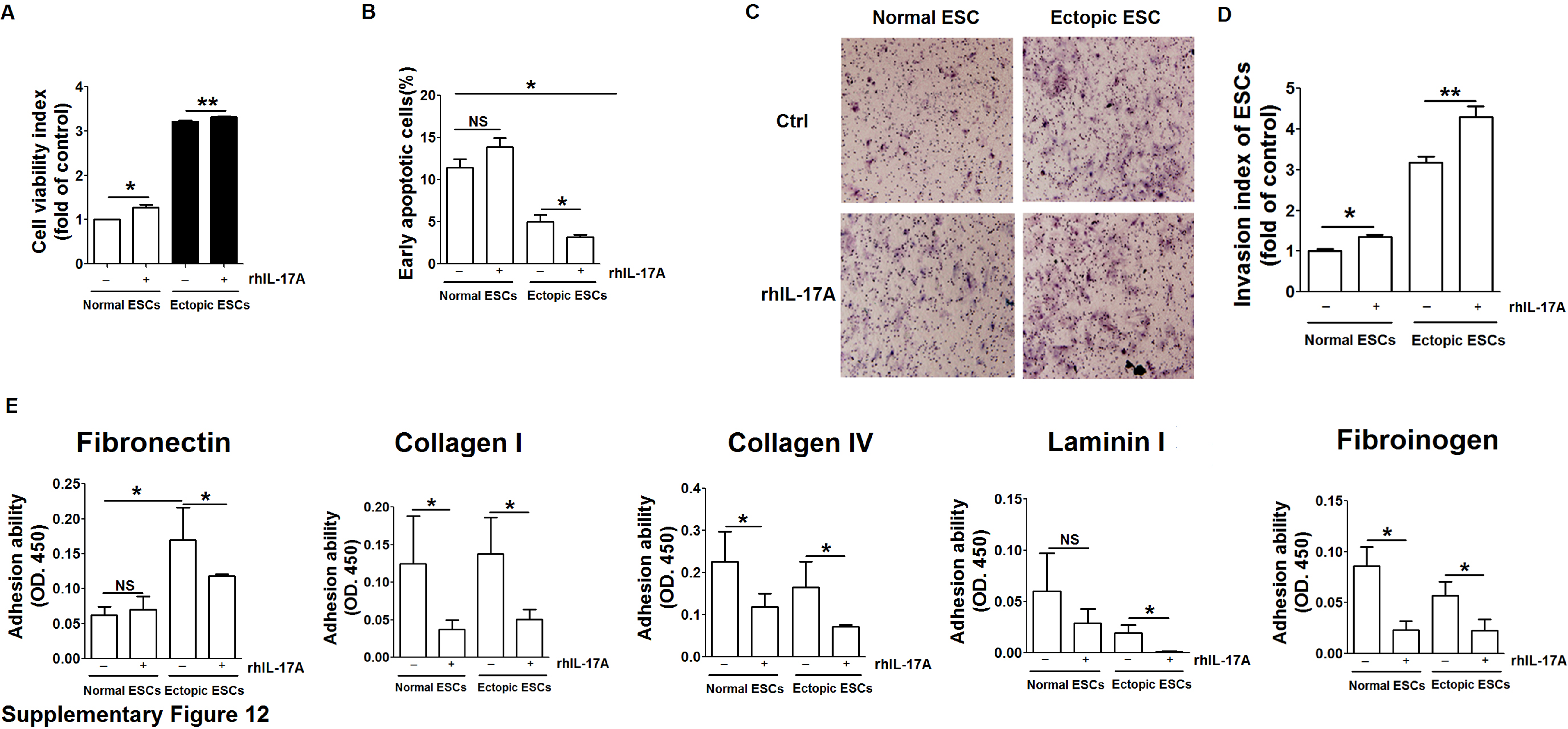

Supplement: Supplementary Figure 12 [file cddis201795x12.tif]

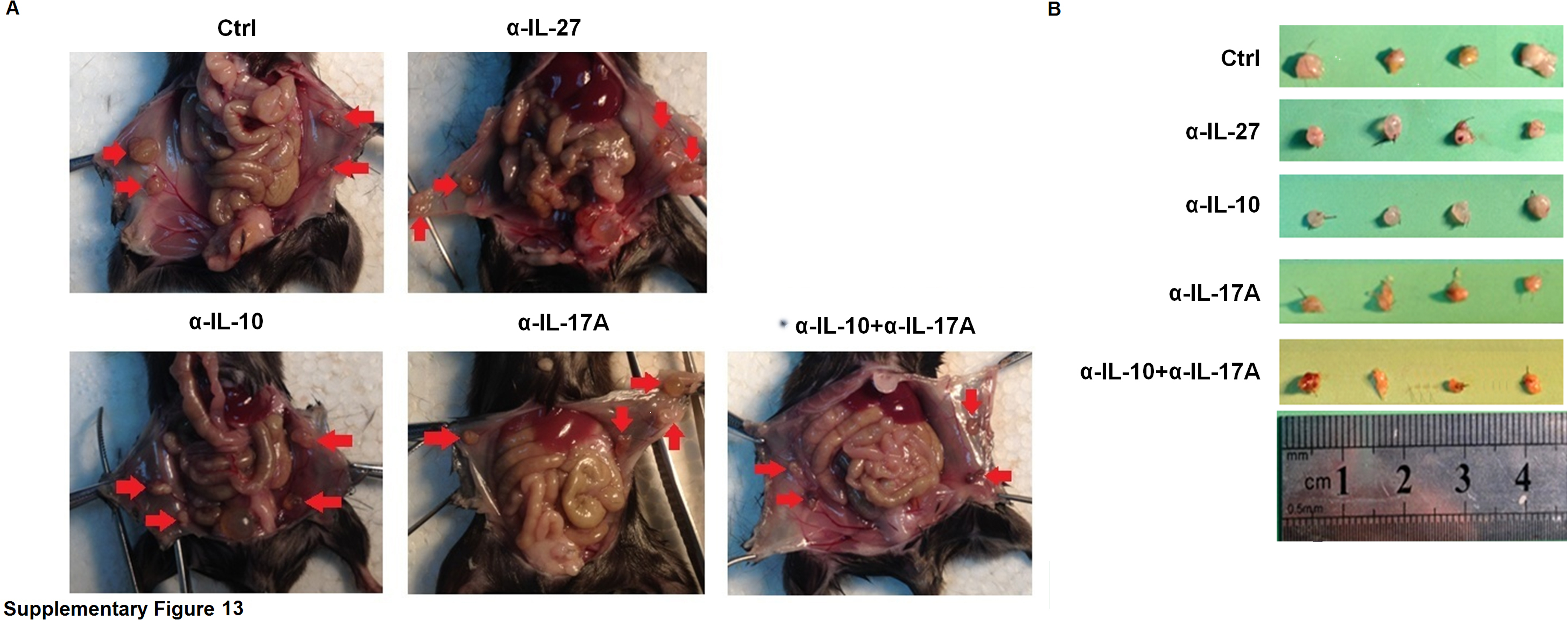

Supplement: Supplementary Figure 13 [file cddis201795x13.tif]

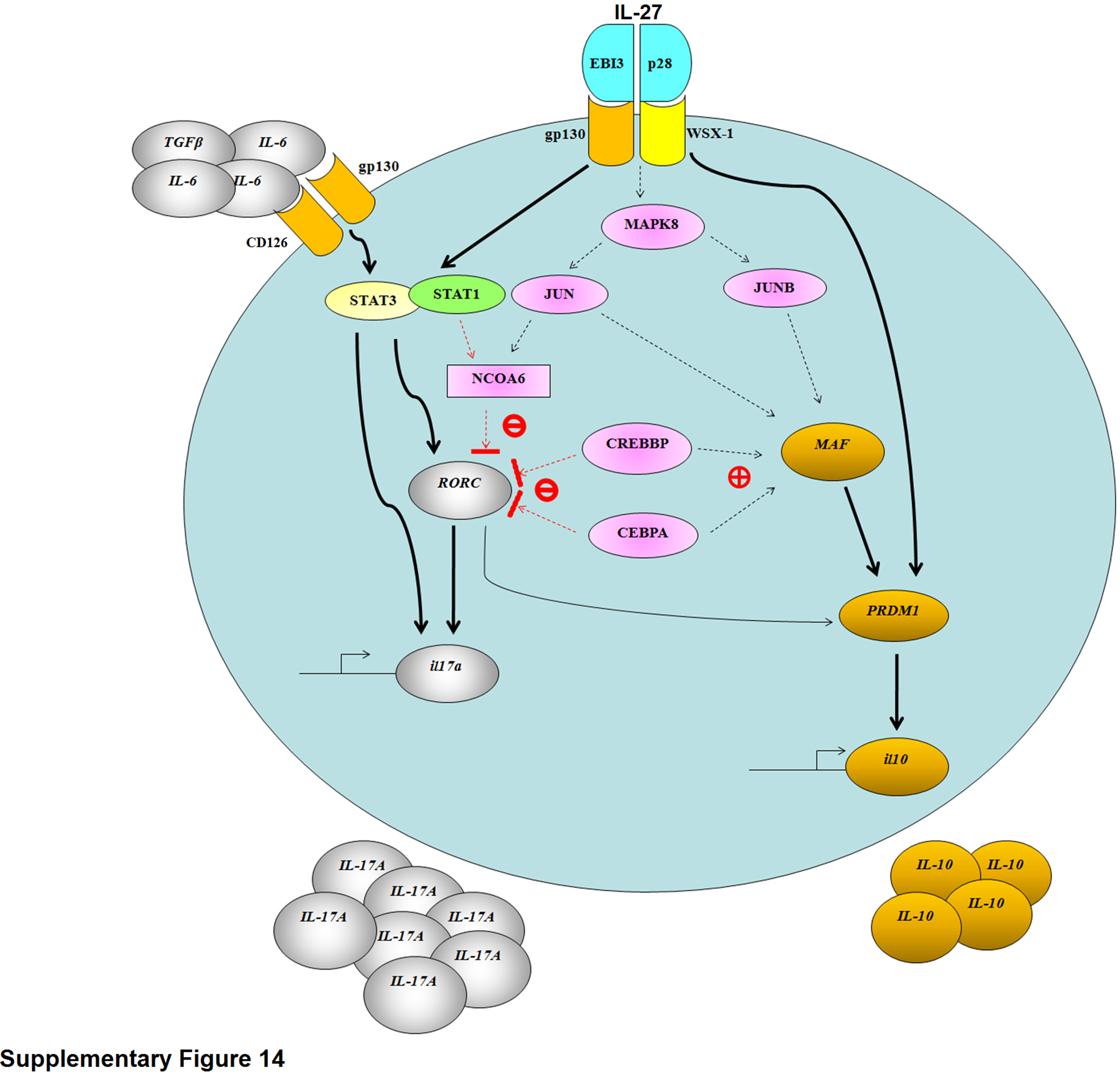

Supplement: Supplementary Figure 14 [file cddis201795x14.tif]
