# Supplementary material for: IL-27 triggers IL-10 production in Th17 cells via a c-Maf/RORγt/Blimp-1 signal to promote the progression of endometriosis
Source: Cell Death Dis. 2017 Mar 16;8(3):e2666–. doi: 10.1038/cddis.2017.95 (PMC5386585; doi:10.1038/cddis.2017.95)
Supplement: Supplementary Information [file cddis201795x15.doc]

**Supplementary Information**

**Antibody for Flow cytometry (FCM)**

To identify and evaluate the ratio, IL-10 and IFN-γ levels of Th17 cells in PF from women with or without endometriosis, and CD4^+^T cells in co-culture system *in vitro*, these cells were stained with fluorescein isothiocyanate (FITC)-conjugated anti-human CD4 (300506, Biolegend), allophycocyanin (APC)-conjugated IL-17A (512334, Biolegend), PE-conjugated Foxp3 (320207, Biolegend), phycoerythrin -cyanine 7 (PE-Cy7)-conjugated IL-10 (510421, Biolegend), PE-conjugated IFN-γ monoclonal (502509, Biolegend) and APC-Cy7-conjugated anti-human CD45 (304014, Biolegend) Abs; To analyze the expression of IL-27 in ESCs and monocytes/macrophages, these cells were stained with APC-conjugated anti-human IL-27 (IC25261A, R&D), Alexa Fluor® 488-conjugated anti-human Vimentin (IC2105G, R&D), and PE-Cy7-conjugated CD14 monoclonal (325618, Biolegend) Abs; To identify the level of IL-27 receptors (WSX-1 and gp130) on human CD4^+^T cells from peripheral blood, normal endometrium and ectopic lesion, the PBMC and lymphocytes in endometrium were stained with APC-Cy7-conjugated anti-human CD45 (304014, Biolegend), PE-Cy7-conjugated CD4 (300512, Biolegend), PE-conjugated WSX-1 (FAB14791P, R&D), APC-conjugated gp130 (FAB228A, R&D), FITC-conjugated IL-17A (512304, Biolegend); FITC-conjugated Foxp3 (320106, Biolegend), FITC-conjugated T-bet (561266, BD) and or FITC-conjugated GATA-3 (560163, BD) Abs; ESCs were stained by FITC-conjugated anti-human Vimentin (IC2105G, R&D), APC-conjugated CD29 (303008, Biolegend) and PE-conjugated CD28 (342104, Biolegend) Abs.

To analyze the level of Th17 cells, IL-10, IFN-γ, Blimp-1, c-Maf and RORγt of PKH26-labled CD4^+^T cells in PF from mice endometriosis model, the lymphocytes in PF were stained with PE-conjugated anti-mouse IL-17A (506904, Biolegend), PE-Cy7-conjugated anti-mouse IL-10 (505026, Biolegend) and Alexa Fluor® 647-conjugated anti-mouse IFN-γ (505814, Biolegend) Abs, Alexa Fluor® 647-conjugated anti-mouse Blimp-1 (563643, Biolegend), Alexa Fluor® 647-conjugated anti-mouse RORγt (562682, BD) and PE-conjugated anti-human/mouse c-Maf Abs (565795, BD) ; To identify the expression of IL-27 in mouse macrophages of liver, spleen and uterus, and uterus stromal cells (USC), these cells were stained with FITC-conjugated anti-mouse CD45 (103108, Biolegend), PE-Cy7-conjugated anti-mouse F4/80 (123114, Biolegend), APC-conjugated IL-27 (IC1834A, R&D) and or FITC-conjugated anti-mouse Vimentin (MBS570210, MyBioSource) Abs; CD4^+^T cells from mouse uterus, spleen and liver were stained by FITC-conjugated mouse CD45 (103108, Biolegend), APC-conjugated mouse CD4 (110412, Biolegend), PE-conjugated mouse WSX-1 (FAB21091P, R&D) and or FITC-conjugated anti-mouse Vimentin (MBS570210, MyBioSource) Abs.

**Collection and preparation of peritoneal fluid (PF)**

PF was aspirated from the *cul de sac* at the beginning of the standard laparoscopic procedure under general anesthesia. Samples of PF contaminated by blood were excluded from the study. The samples were centrifuged at 1500 rpm for 10 min, and then frozen at -80°C. The IL-27 (BioLegend) concentration was determined using ELISA kits.

The mononuclear cells were isolated from the PF using lymphocyte separation medium and density-gradient centrifugation at 2000 rpm for 20 min at 4°C. The cells were collected, washed in ice-cold PBS and suspended in antibody staining buffer at the desired concentration for flow cytometry.

**Isolation and culture of primary ESC**

All the endometrial tissues (health controls) and endometriotic lesion tissues were collected under sterile conditions and transported to the laboratory on ice in DMEM (Dulbecco’s modified Eagle’s medium)/F-12 (Gibco, USA) with 10% fetal calf serum (FCS; Hyclone, Logan, UT, USA). The endometrial tissues were digested with collagenase type IV (0.1%; Sigma, USA) for 30 min at 37 °C with constant agitation for recovering ESCs. The tissue pieces were filtrated through sterile gauzes (pore diameter sizes: 200 mesh) to remove debris. Following gentle centrifugation, the supernatant was discarded, and the cells were resuspended in DMEM/F-12. The ESCs were separated from epithelial cells by passing them over sterile gauzes (pore diameter sizes: 400 mesh). The filtrated suspension was layered over Ficoll, and centrifuged at 800×g for 20 min to further remove leukocytes and erythrocytes, and the middle layer was collected, and then washed with D-Hanks solution. The ESCs were placed in a culture flask, and allowed to adhere for 20 min. The adherent stromal cells were cultured as monolayer in flasks with DMEM/F-12 containing 10% FCS, and incubated in 5% CO_2_ at 37 °C. This method supplied a 95% vimentin-postive and cytokeratin-negative ESCs.

The HEK-293T cell line (purchased from Bank of Cell, Chinese Academy of Sciences, Shanghai, China) was maintained in RPMI 1640 medium with 10% FCS.

**Immunohistochemistry**

Paraffin sections (5 um) of the endometriosis-like lesions and normal endometrium were dehydrated in graded ethanol, then incubated with hydrogen peroxide and 1% bovine serum albumin (BSA)/TBS to block endogenous peroxidase. The samples were then incubated with mouse anti-human IL-27 (25 µg/ml, R&D) or mouse IgG isotype overnight at 4°C in a humid chamber. After washing three times with TBS, the sections were overlaid with peroxidase-conjugated goat anti-mouse IgG, and the reaction was developed with 3,3-diaminobenzidine (DAB) and counterstained with hematoxylin.

**Plasmids**

The information about overexpression plasmids and luciferase reporter plasmids was shown in Supplementary Figure 11. All plasmids were from GeneChem Co., Ltd (Shanghai, China).

**Cell viability and apoptosis assays**

The Cell Counting Kit-8 (CCK-8) (Dojindo, Japan) assay and Annexin V-FITC (Invitrogen, USA) assay were applied to evaluate the effects of IL-10, IL-17A and Th17 cells on cell viability and apoptosis of ESCs, respectively. ESCs were re-suspended in DMEM/F-12 with 10% FBS, and seeded at a density of 1×10^4^ cells/well in 96-well flat-bottom microplates (for CCK-8 assay), or 1×10^5^ cells/well in 24-well flat-bottom microplates (for Annexin V-FITC assay). After 70–80% confluence, the cells were starved with DMEM containing 1% FBS for 12 h before treatment. The medium was removed once again, and the cells were treated with recombinant human (rh) IL-17A (10 ng/ml), rhIL-10 (100ng/ml) or Th17 cells, respectively, with vehicle as control.

According to the manufacturer’s protocol, the CCK8 reagent was added to each well and cells were incubated at 37°C for 1-4 h. The absorbance (optical density) at 450 nm was measured and used to represent the viability of cells. Each experiment was performed in six parallel wells, and repeated three times.

Phosphatidylserine externalization was quantified by flow cytometry by using a commercially available annexin V-FITC apoptosis detection kit (Invitrogen, USA) according to the manufacturer’s guideline. ESCs were trypsinized and collected. The culture medium was also retained and pooled with the adherent cells. Cells were centrifuged, and the supernatant was discarded. The cells were resuspended in PBS, and washed twice and resuspended in the kit binding buffer (100 ml/pellet) containing annexin V solution (5 ul/pellet) and propidium iodide (2.5 mg/ml). Samples were incubated in dark for 15 min, and the percent of annexin V-positive cells in ESCs was determined by FACSCalibur flow cytometry. The experiments were performed in triplicate, and repeated three times.

**Matrigel invasion assay**

The invasion of the ESCs across Matrigel (BD) was evaluated objectively in invasion chamber. Briefly, the cells inserts (8um pore size, 6.5 mm diameter, Corning, USA) coated with 15-25 ul Matrigel were placed in a 24-well plate. The primary ESCs of 2×10^4^ were plated in the upper chamber (the media contained 1% charcoal stripped FCS). RhIL-10 and or rhIL-17A were added, respectively. The lower chamber (the media was contained 5% charcoal stripped FCS) was filled with 800ul medium. The cells were then incubated at 37 °C for 48 h. The inserts were removed, washed in PBS, and the noninvading cells together with the Matrigel were removed from the upper surface of the filter by wiping with a cotton bud. The inserts were then fixed in methanol for 10 min at room temperature and stained with hematoxylin. The result was observed under Olympus BX51+DP70 microscope (Olympus, Tokyo, Japan). The cells migrated to the lower surfaces were counted at a magnification of x200. The cells migrated to the lower surfaces were counted in five predetermined fields. Each experiment was carried out in triplicate, and repeated three times.

**Intraperitoneal endometriosis model**

For C57BL/6 (8 weeks olds) mice, the intraperitoneal endometriosis-like lesions were induced surgically by suturing uterine tissue samples to the abdominal wall. For autologous transplantation, the left uterine of the recipient animal was divided into 4 equal parts, and sewn into four quadrants of the peritoneum. Endometriosis-like lesions developed along the transplanted uterine tissue samples. The mice were treated with anti-mouse IL-27 neutralizing Abs (αIL-27, 50 µg/mouse), αIL-10 (50 µg/mouse) or αIL-17A (50 µg/mouse), recombinant mouse IL-27-Fc protein (rmIL-27-Fc, 40ng/mouse) and or αIL-2 (50 µg/mouse) every week after surgery. After two weeks, the size of endometriosis-like lesions was detected.

For nude (8 weeks olds) mice, we constructed an allotransplantation of intraperitoneal endometriosis model. On Day 0, the uterus of female C57BL/6 mice was minced, then the tissue debris was intraperitoneal injected to nude mice (the ratio of uterus to intraperitoneal injection of nude mice was 1:2). On Day 5, the isolated WSX-1^-^CD4^+^ T cells or WSX-1^+^CD4^+^ T cells from uterus of female C57BL/6 mice was labeled with PKH-67 (Sigma) and transferred to the abdominal cavity in endometriosis nude mice. On Day 14, the endometriosis-like lesions and PF were collected and detected. Then, we collected these cells in PF and evaluated Th17 differentiation and relative molecules expression by flow cytometry. In addition, immunohistochemistry was used to analyze the expression of Ki-67 (15ug/ml, R&D) and MMP2 (25ug/ml, R&D) in the endometriosis-like lesions.

**Supplementary Methods**

**Reagents.** Anti-human CK7 and Vimentin Abs were purchased from ZSGB-BIO (ZSGB, Beijing, China); Cytokine Cytometric Bead Array (CBA) Kit, and matrigel were purchased by BD Biosciences (USA); Bio-Plex Suspension Array was from Bio-Rad Laboratories, Inc; Human IL-27 ELISA Kit was from Biolegend (Cat. No. 434607, LEGEND MAX™); The CCK8 kit was purchased from DOJINDO (Shanghai, China), The adhesion assay kits was from Cell Biolabs (San Diego, CA, USA) and LPS and PGE2 were from Sigma (USA).

**Flow cytometry.** Flow cytometry was performed to analyze: 1) the MFI of cytokines (IFN-γ, TNF-α, IL-1β, IL-17A, IL-6, IL-21, IL-2, TGF-β, IL-10 and IL-4) (CBA assay); 2) the percentage of Th17 cells and CD14^+^macrophages in PF from women with or without endometriosis and CD4^+^T cells in co-culture system *in vitro*; 3) IL-27 level in macrophages from spleen, liver and uterus, and USC, 4) IL-27 receptors on CD4^+^T cells from spleen, liver and uterus; 5) c-Maf level in human CD4^+^T cells; 6) IL-2 in macrophages. The samples were analyzed using a FACS Calibur flow cytometer (Becton Dickinson, USA) and Cellquest software (Becton Dickinson). The statistical analysis was conducted by using isotype matched controls.

**Immunocytochemical staining (ICC).** For ICC, ESCs growing on coverslips were cultured for 48h. The coverslips were fixed in 4% (vol/vol) paraformaldehyde for 20 min at room temperature, washed in PBS and permeabilized for 10 min with 0.25% (vol/vol) Triton-100 in PBS. The cells were then incubated with 1% BSA in PBS/Tween (PBST) for 30 min to block non-specific binding of antibodies. The anti-human vimentin monoclonal antibody as markers for ESCs, and cytokeratin-7 antibodies as markers for epithelial cells were then added. The cells were incubated with primary antibody or isotypic control overnight at 4°C, and then incubated with a peroxidase-conjugated secondary antibody for 60 min at 37°C. The slides were stained with DAB, and counterstained with haematoxylin. The experiments were repeated three times.

**ELISA.** ESCs from healthy endometrium, eutopic endometrium and ectopic lesions from women with endometriosis were cultured in 24-well plates for 48 h (1×10^5^ cells/500ul/well). These ESCs (1×10^5^ cells/500ul/well) were cultured with peripheral blood monocytes (1×10^5^ cells/500ul/well) for 48 h. ESCs alone and monocytes alone were included as controls. In addition, monocytes were incubated with LPS (10ng/ml) or PGE2 (10^-6^M). Then the cell culture supernatant was assayed by ELISA or Bio-Plex Suspension Array to determine the secretion levels of IL-27, IL-6, TGF-β, MCP-1, RANTES, GM-CSF, TNF-α, IL-1β and IL-10.

**CCK8 assay, Annexin V-FITC assay, Matrigel invasion assay and Adhesion assay.** After treatment with or without rhIL-17A (10ng/ml), the level of viability, apoptosis, invasion and the ability for adhesion to extracellular matrix (ECM) in ESCs were measured by CCK-8 assay, annexin V-FITC apoptosis detection kit, matrigel invasion assay and adhesion assay, respectively.

**Supplementary Figure legends**

**Supplementary Figure 1: The cytokine profile in PF from women with endometriosis.** The MFI of cytokines (IFN-γ, TNF-α, IL-1β, IL-17A, IL-6, IL-21, IL-2, TGF-β, IL-10 and IL-4) in PF from women with or without enodmetriosis were measured by CBA assay. Ctrl: PF from women without endometriosis (n=6); EMS S(I-II): PF from women with endometriosis were in early stages (stage I and II, n=8); EMS S(III-IV): PF from women with endometriosis were in advanced stages (stage III and IV, n=8). Data are expressed as the mean±SEM. **P*<0.05 and ***P*<0.01. NS: no statistically difference. **(**One-way ANOVA)

**Supplementary Figure 2: The level changes of CD4^+^T cells, Th17 cells and macrophages in PF from women with endometriosis. (A-B)** The percentage of CD4^+^T cells in total CD45^+^ cells, Th17 cells in CD4^+^T cells, CD14^+^macrophages in total CD45^+^ cells, and the ratio of CD14^high^ to CD14^low^ and CD14^high^CD16^+^ to total CD14^+^macrophages in PF from women with (n=8) or without (n=6) endometriosis by flow cytometry. Data are expressed as the mean±SEM. ***P*<0.01 and ****P*<0.001. **(**One-way ANOVA)

**Supplementary Figure 3: The characterization of primary ESCs.** The primary isolated ESCs (n=3/group) from normal endometrium (Normal ESCs), eutopic endometrium (Eutopic ESCs) and ectopic lesion (Ectopic ESCs) with endometriosis was identified by ICC. Immunocytochemistry showed >95% vimentin-positive and cytokeratin-negative ESCs. Original magnification: ×200.

**Supplementary Figure 4: Co-culture of ESC and monocytes leads to high level of cytokines.** We co-cultured ESCs (normal ESCs, eutopic ESCs or ectopic ESCs, n=5) with monocytes (n=5) from peripheral blood for 48h. In addition, monocytes were stimulated with LPS (10ng/ml) or PGE2 (10^-6^M), and ESCs alone and monocytes alone were cultured as control. Then the secretion level of IL-27, IL-6, TGF-β, MCP-1, RANTES, GM-CSF, TNF-α, IL-1β and IL-10 was analyzed by ELISA. Normal ESCs: ESCs from normal endometrium; eutopic ESCs: ESCs from eutopic endometrium with endometriosis; ectopic ESCs: ectopic lesion with endometriosis. Data are expressed as the mean±SEM. **P*<0.05, ***P*<0.01 and ****P*<0.001. **(**One-way ANOVA)

**Supplementary Figure 5: Macrophage and stromal cell from mouse uterus high express IL-27.** The expression of IL-27 in macrophages (**A-C**) from C57BL/6 mice (n=5) liver, spleen, uterus and uterus stromal cells (USC) (**B,C**) was analyzed by flow cytometry. Data are expressed as the mean±SEM. ***P*<0.01 and ****P*<0.001. **(**One-way ANOVA)

**Supplementary Figure 6: IL-10^+^Th17 cells in ectopic lesion high express IL-27R.** (**A**) The expression of IL-27R (WSX-1 and gp130) on CD45^+^CD4^+^T cells from human peripheral blood (pCD4^+^T) (n=6), normal endometrium (nECD4^+^T) (n=5) and ectopic lesion (eECD4^+^T) (n=5) by flow cytometry. (**B, C**) IL-27R expression on CD4^+^T-bet^+^Th1 cells, CD4^+^GATA-3^+^Th2 cells, CD4^+^Foxp3^+^Treg cells and CD4^+^IL-17A^+^Th17 cells in CD45^+^mononuclear lymphocytes from human peripheral blood (PBMC), normal endometrium (nELC) and ectopic lesion (eELC) by flow cytometry. (**D**) IL-27R expression on IL-10^+^Th17 cells and IL-10^-^Th17 cells in CD45^+^mononuclear lymphocytes from ectopic lesion by flow cytometry. Data are expressed as the mean±SEM. **P*<0.05, ***P*<0.01 and ****P*<0.001. **(**One-way ANOVA)

**Supplementary Figure 7: IL-27 triggers IL-10 produce of Th17 cells induced by IL-6 and TGF-β *in vitro*.** (**A**) The human naïve T cells (n=5) from peripheral blood were stimulated with rhIL-6 (50ng/ml), TGF-β (2ng/ml) and or IL-27 (100ng/ml) for 5 days, then Th17 differentiation, IL-10 and IFN-γ levels in Th17 cells were detected by flow cytometry. (**B**) After stimulation with rhIL-6 and or rhIL-27, the level of PD-L1 on CD4^+^T cells was analyzed by flow cytometry. (**C,D**) Mouse naïve T cells (n=5) were differentiated to Th17 cells and stimulated with or without rhIL-27 (25ng/ml) *in vitro* for 5 days. Then the level of Th17 cells and IL-10 level in Th17 cells was analyzed by flow cytometry. Data are expressed as the mean±SEM. **P*<0.05, ***P*<0.01 and ****P*<0.001; ^#^*P*<0.05 and ^###^*P*<0.001 vs. Th17 group; ^$$^*P*<0.01 and ^$$$^*P*<0.001 vs. Th0 plus IL-27 group. **(**One-way ANOVA)

**Supplementary Figure 8: IL-27 is not involved in regulating the differentiation and IL-10 produce of Treg *in vitro*.** (**A,B**) After co-culture of ESCs and monocytes for 48h, the monocytes were collected and added to the human naïve T cells (n=5) from peripheral blood, and further cultured for 5 days, then Treg cells differentiation, IL-10 levels in Treg cells were detected by flow cytometry. Data are expressed as the mean±SEM. ****P*<0.001. (One-way ANOVA)

**Supplementary Figure 9: CD4^+^T cells from mouse uterus high express WSX-1.** (**A,B**) The expression of WSX-1 on CD4^+^T cells from the C57BL/6 mice (n=5) liver, spleen, uterus by flow cytometry. Data are expressed as the mean±SEM. ****P*<0.001. (One-way ANOVA)

**Supplementary Figure 10: IL-10^+^Th17 cells high express c-Maf.** The human naïve T cells (n=5) from peripheral blood were differentiated to Th1, Th2, Treg and Th17 cells *in vitro*. for 5 days, then c-Maf in CD4^+^T-bet^+^Th1 cells, CD4^+^GATA-3^+^Th2 cells, CD4^+^Foxp3^+^Treg cells and CD4^+^IL-10^+^IL-17A^+^Th17 cells and CD4^+^IL-10^-^IL-17A^+^Th17 cells were detected by flow cytometry. Data are expressed as the mean±SEM. ****P*<0.001. (One-way ANOVA)

**Supplementary Figure 11: The information for plasmids, the procedure of Dual luciferase reporter assay and primer sequences.** (**A**) The information of overexpression plasmids and luciferase reporter plasmids for Dual luciferase reporter assay. (B) The characterization of IL-27 overexpression in HEK 293T after transfection by Olympus BX51 fluorescence microscope (left) and ELISA (right). (C) The procedure **o**f Dual luciferase reporter assay: HEK 293T cells were transfected with empty plasmid or recombinant plasmid. Transient transfection experiments were performed in 24-well plates using 0.4μg empty plasmid or recombinant plasmid, in combination with PGL-3 reporter vector or pGL-3-gene reporter vector. And pTR-TK transfection was considered as internal control. **(D)** The primer sequences for *PRDM1*, *MAF*, *RORc*, *IL-10* and *GAPDH*. Data are expressed as the mean±SEM. ***P*<0.01. (One-way ANOVA)

**Supplementary Figure 12: IL-17A promotes viability and invasion, and restricts apoptosis and adhesion of ectopic ESCs.** The normal ESCs and ectopic ESCs were stimulated with or without rhIL-17A (10ng/ml) for 48h, then the cell viability (A), apoptosis (B), invasion (C,D) and adhesion to ECM (Fibronectin, Collagen I, Collagen IV, Laminin I and Fibroinogen) (E) were analyzed by CCK-8 assay, apoptosis assay, matrigel invasion assay and adhesion assay. Data are expressed as the mean±SEM. **P*<0.05 and ***P*<0.01. (Student’s *t*-test).

**Supplementary Figure 13:** **IL-27 promotes growth of mouse endometriosis-like lesion *in vivo***. (**A, B)** The size of endometriosis-like lesions from C57BL/6 endometriosis mice model was measured after treatment with αIL-27 (50 µg/mouse), αIL-10 (50 µg/mouse) and or αIL-17A (50 µg/mouse).

**Supplementary Figure 14: The** **possible signal net of IL-27 in stimulating IL-10 production of Th17 cells differentiation induced by IL-6 and** **TGF-β**. A network picture about IL-27 was drawn based on our research project and several databases, including Pathway Commons Project, KEGG, Pathway Interaction Database and IntAct Database. Under the stimulation of IL-6 and TGF-β, the STAT3 is activated in naïve T cells, which further promotes *RORc* and *IL-17A* transcription, and induces the differentiation of Th17 cells and IL-17A produce. If IL-27 also co-exists in this environment, the interaction of IL-27 (EBI3 and p28) and receptors (WSX-1 and gp130) possibly activates AP-1 family transcription factors (such as proteins JUN and JUNB), which further induce PRDM1 and down-stream IL-10 transcription by inhibiting *RORc* and promoting *MAF*, finally triggers IL-10 produce of Th17 cells.
